# Metabolic pathways fueling human spermatozoa capacitation and hyperactivation: a roadmap for spermatozoa bioenergetics

**DOI:** 10.3389/fendo.2026.1882458

**Published:** 2026-07-22

**Authors:** Eva V. Gonçalves, David F. Carrageta, Bárbara Guerra-Carvalho, Mário Sousa, Raquel L. Bernardino

**Affiliations:** 1 Laboratory of Cell Biology, Department of Microscopy, Unit for Multidisciplinary Research in Biomedicine, School of Medicine and Biomedical Sciences, University of Porto, Porto, Portugal; 2 Unit for Multidisciplinary Research in Biomedicine, School of Medicine and Biomedical Sciences, University of Porto, Porto, Portugal; 3 ITR - Laboratory for Integrative and Translational Research in Population Health, Porto, Portugal; 4 LAQV-REQUIMTE, Department of Chemistry, University of Aveiro, Aveiro, Portugal

**Keywords:** bioenergetics, capacitation, hyperactivation, hypermotility, male fertility, metabolic pathways, motility, spermatozoa

## Abstract

Human spermatozoa are highly specialized cells whose function depends on tightly regulated metabolic and signaling networks. Following spermatogenesis and epididymal maturation, ejaculated spermatozoa must undergo capacitation in the female reproductive tract, a complex process characterized by extensive biochemical remodeling and high energy demand that enables hyperactivated motility and fertilizing competence. Human spermatozoa bioenergetics remains a prominent theme in the field of Andrology, whose advances are challenged by inter-species differences and difficulties in extrapolating data from *in vitro* and animal model studies to human physiology. In this comprehensive review, we discuss the current knowledge of the bioenergetic pathways governing capacitation and hyperactivation in human spermatozoa. While glycolysis remains a primary adenosine triphosphate (ATP) source, supported by sperm-specific glycolytic enzymes that provide a rapid and spatially localized ATP supply directly in the flagellum, accumulating evidence indicates that mitochondrial oxidative phosphorylation (OXPHOS) complements ATP production, assists in the regeneration of reducing equivalents to sustain a high glycolytic flux during capacitation and contributes to capacitation-activating signaling pathways. Besides these core bioenergetic pathways, the great metabolic flexibility of human spermatozoa is evidenced by the mobilization of endogenous energy substrates and engagement of additional metabolic pathways, such as fatty acid β-oxidation, pentose phosphate pathway, ketone body catabolism, and amino acids oxidation to meet the high energetic and redox demands required for capacitation and hyperactivation. Overall, these findings suggest that the metabolic potential of human spermatozoa is considerably broader than previously recognized. A comprehensive understanding of human spermatozoa bioenergetics will assist in the identification of novel biomarkers that may characterize previously male infertility cases identified as idiopathic, as well as identify therapeutic targets to improve medically assisted reproductive procedures.

## 1 Introduction

The development of competent male gametes, the spermatozoa, is a complex, precise and tightly regulated process that depends on coordinated autocrine, paracrine, and endocrine interactions among testicular cells (Sertoli, Leydig, and germ cells), epididymal cells, and the hypothalamus-pituitary axis (1, 2). Spermatogenesis is the biological process that produces spermatozoa from spermatogonial stem cells in the seminiferous tubules of the testes. During spermatogenesis, a critical balance between the self-renewal and differentiation of spermatogonial stem cells is maintained to ensure the continuous production and release of spermatozoa (3). Spermiation is the last step of spermatogenesis, where spermatozoa are released to the lumen of the seminiferous tubules in an immature and immotile state, requiring to be transported, through seminiferous fluid dynamics and peritubular myoid cell peristaltic movements, into the epididymis. The epididymis is structurally divided into three regions: the head (*caput*), an initial enlarged region in continuity with the testis through the *rete testes* and efferent ducts; a central region, referred to as the body (*corpus*); and the tail (*cauda*), the most distal region that connects to the *vas deferens* (4). The epididymis has a double role: firstly, the coordination of a maturation step that occurs in the *caput* and *corpus*, responsible for the spermatozoa’s final deoxyribonucleic acid (DNA) condensation and acquisition of motility; secondly, the maintenance of a spermatozoa’s reservoir in the *cauda*, where the male gametes are kept in a quiescent but viable state until either ejaculation or degeneration (5). The steps of epididymal maturation are strongly influenced by the epididymal microenvironment, which is shaped by the epididymal fluid and epididymosomes, small vesicles released by the epididymal epithelium that are composed of nucleic acids, metabolites, proteins, glycoproteins, lipids, enzymes, and ions, that induce the biochemical and morphological changes required for spermatozoa maturation, as extensively described in the literature (5–7).

Even though spermatozoa are motile after the epididymal maturation steps, the male gamete is not entirely ready for fertilization. Once ejaculated in the vagina, spermatozoa are selected based on their morphology and motility during the transit through the cervical canal. Those that reach the uterine cavity contact the endometrium, where interactions with the endometrial microenvironment trigger a new series of structural and biochemical modifications. These modifications are collectively denominated as capacitation, a mandatory step before oocyte fertilization (8, 9). Capacitation is broadly defined as the functional modifications that make spermatozoa achieve a hyperactivation state and acquire competence for fertilization, characterized by a change in the movement pattern from a symmetric beat to an asymmetrical and vigorous flagellar beat with greater amplitude in its curvature (9). These changes include membrane remodeling towards an increased fluidity, modulation of enzymatic activity, increased oxidative metabolism, and activation of signaling pathways that lead to the acquisition of hyperactivated motility, chemoattractant responsiveness, the ability to traverse the extracellular matrix of cumulus cells and bind to the oocyte *zona pellucida* (ZP), undergo the acrosome reaction, and, finally, fuse with the oocyte membrane (10).

**Figure 1:**
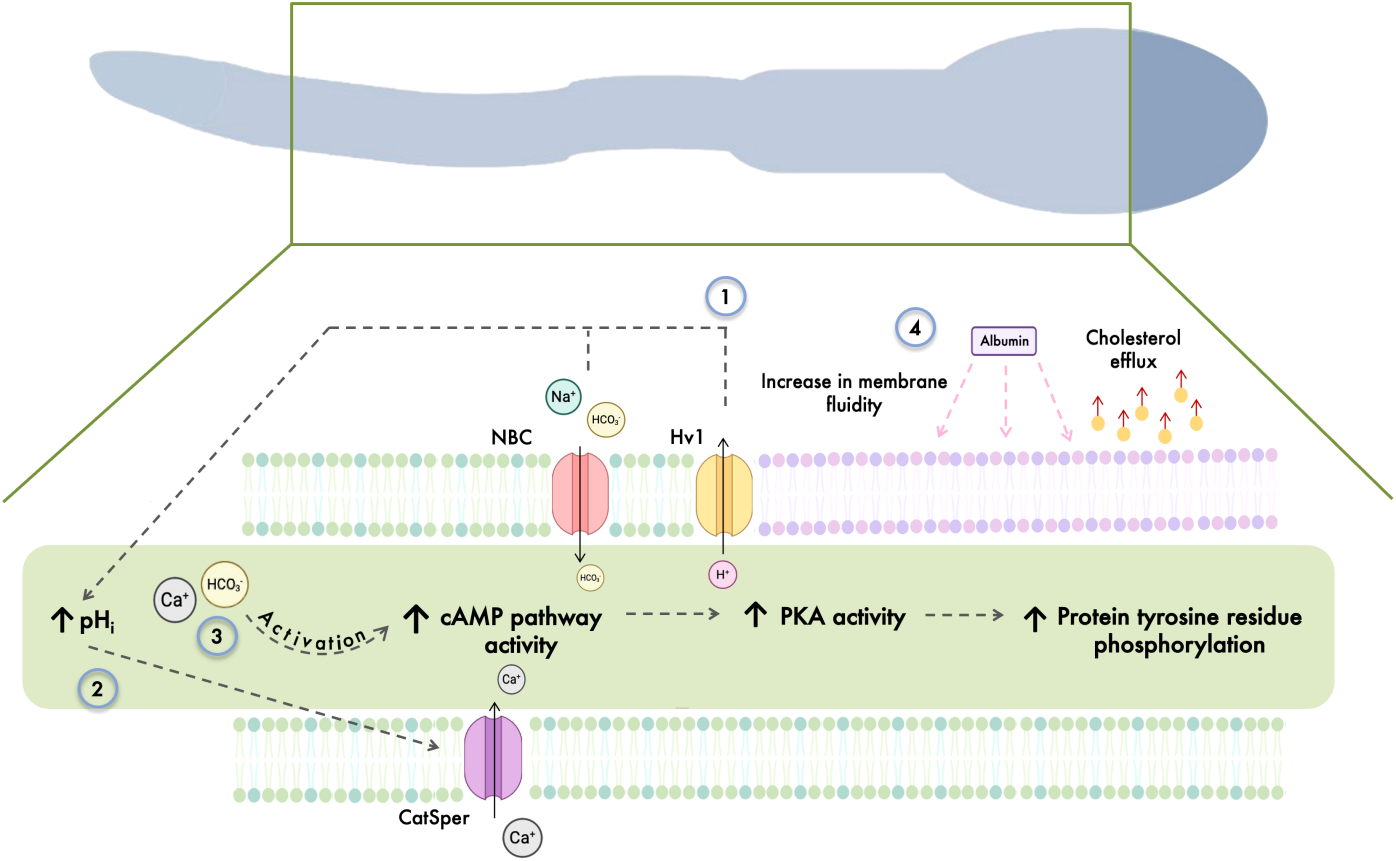
Molecular and ionic mechanisms underlying human spermatozoa capacitation. Human spermatozoa capacitation is a complex biochemical and physiological process that occurs in the female reproductive tract that enables spermatozoa to acquire fertilizing competence. (1) Capacitation is initiated by the influx of bicarbonate (HCO_3_^-^) through sodium-bicarbonate cotransporters (NBC), leading to intracellular alkalinization. This rise in pH is reinforced by proton (H^+^) efflux via voltage-gated proton channels (Hv1), particularly in the principal piece of the flagellum. (2) Increased intracellular pH (pHi) activates the spermatozoa cation channel (CatSper), promoting Ca^2+^ entry (3) which together with HCO_3_^-^ stimulates soluble adenylyl cyclase (sAC), elevating intracellular cyclic AMP (cAMP) levels and activating protein kinase A (PKA). PKA signaling triggers a sperm-specific tyrosine phosphorylation cascade, resulting in extensive phosphorylation of flagellar proteins. (4) Simultaneously, albumin present in the female reproductive tract extracts cholesterol from the spermatozoa plasma membrane, increasing membrane fluidity. These events drive membrane reorganization and the transition from progressive motility to hyperactivated motility, both essential for successful fertilization.

Although still debated, capacitation, depicted in **Figure 1**, is thought to be triggered by the influx of HCO_3_^−^ via sodium bicarbonate cotransporters (NBC). The influx of HCO_3_^−^, concomitant with the efflux of protons through voltage-gated proton channels (Hv1) localized in the principal piece of the flagellum membrane, increases the intracellular pH (pH_i_) and subsequently alkalizes the cytoplasm. Capacitation triggers are only activated when spermatozoa are no longer exposed to the decapacitating factors present in seminal fluid, including a high concentration of Zn^2+^, semenogelin, and certain glycoproteins (11, 12). Then, the cytoplasm alkalinization promotes the activation of the Ca^2+^ permeable spermatozoa cation channel (CatSper), which spikes Ca^2+^ influx. Both Ca^2+^ and HCO_3_^−^ influxes induce the activation of the cyclic AMP (cAMP) pathway, ultimately activating several signaling cascades (10). Most of these effects are thought to be mediated by protein kinase A (PKA), which induces tyrosine residues phosphorylation in flagellar proteins by activating downstream tyrosine kinases (10). This process is denominated as the tyrosine phosphorylation cascade, a pathway unique to spermatozoa known as the hallmark of capacitation and hyperactivation. In humans, the tyrosine residues phosphorylation cascade mainly occurs in the fibrous sheath of the flagellum, where A-kinase anchoring proteins (AKAPs) are localized (13). Additionally, and simultaneously to all these processes, the high amounts of albumin in the endometrial and oviduct microenvironments act both as a chelator of Zn²^+^ and as an acceptor of cholesterol and other lipids from the spermatozoa membrane, promoting their efflux and thereby increasing membrane fluidity (10). Only upon these steps can spermatozoa achieve hyperactivation and acquire competence for fertilization.

Human spermatozoa capacitation and hyperactivation require a large adenosine triphosphate (ATP) supply, not only for fueling the increased motility state but also to support capacitation-related enzymatic and signaling processes. Spermatozoa bioenergetics are central for their fertilizing ability; however, data on preferential energy pathways and their regulatory processes remain conflicting. Aside from the high complexity and challenges of studying spermatozoa while recreating, as closely as possible, the human physiological conditions, comparisons with other mammalian species are problematic due to species-specific traits (14, 15). In humans, glycolysis is traditionally recognized as the primary energy pathway driving human spermatozoa’s capacitation and hyperactivation; however, recent evidence suggests that mitochondrial oxidative phosphorylation (OXPHOS) may be equally important, sustaining additional functions beyond ATP production. Moreover, recent studies show that human spermatozoa can use additional metabolic pathways to fuel their energetic and redox needs, implying that human spermatozoa bioenergetics may be more complex than the linear glycolytic-OXPHOS axis initially proposed. In this review, we will explore the most recent data on human spermatozoa bioenergetics, focusing on the metabolic pathways that fuel the ATP production required for capacitation and hyperactivation. Additionally, we will discuss novel evidence on alternative metabolic pathways that may support energy production in human spermatozoa, either relevant for physiological conditions or for the design of future *in vitro* studies. For that purpose, a literature search was conducted using the SCOPUS and PubMed databases, using topic-driven keyword combinations tailored to each thematic section of the review. This exploratory approach, applicable for the narrative nature of this review, allowed flexibility in capturing relevant literature across the multiple biological dimensions addressed. No temporal restrictions were applied, allowing retrieval of all available literature regardless of publication date and until January 2026. Original research articles reporting human data or comparative studies involving human and other mammalian species, with full text availability and published in English, were included in the analysis. Studies were excluded if they were not available in full text, not published in English, or if their conclusions were not substantiated by the presented data. Animal studies were considered exclusively in the context of mechanistic insight or direct physiological comparison with human data and were not used as primary evidence. Priority was given to original research articles; however, relevant review articles were also considered when appropriate to provide broader context. If contradictory findings were identified across studies, these were critically discussed.

## 2 Glycolysis and oxidative phosphorylation: the main metabolic pathways for ATP production in human spermatozoa

Spermatozoa require energy for multiple physiological functions, including the support of their motility and the tyrosine residues phosphorylation cascade that maintains their vitality and capacitated state. Altered metabolic pathways in spermatozoa are commonly associated with infertility cases, including asthenozoospermia (low motility) (16–18); therefore, understanding spermatozoa bioenergetics is among the top priorities in Andrology research.

**Figure 2:**
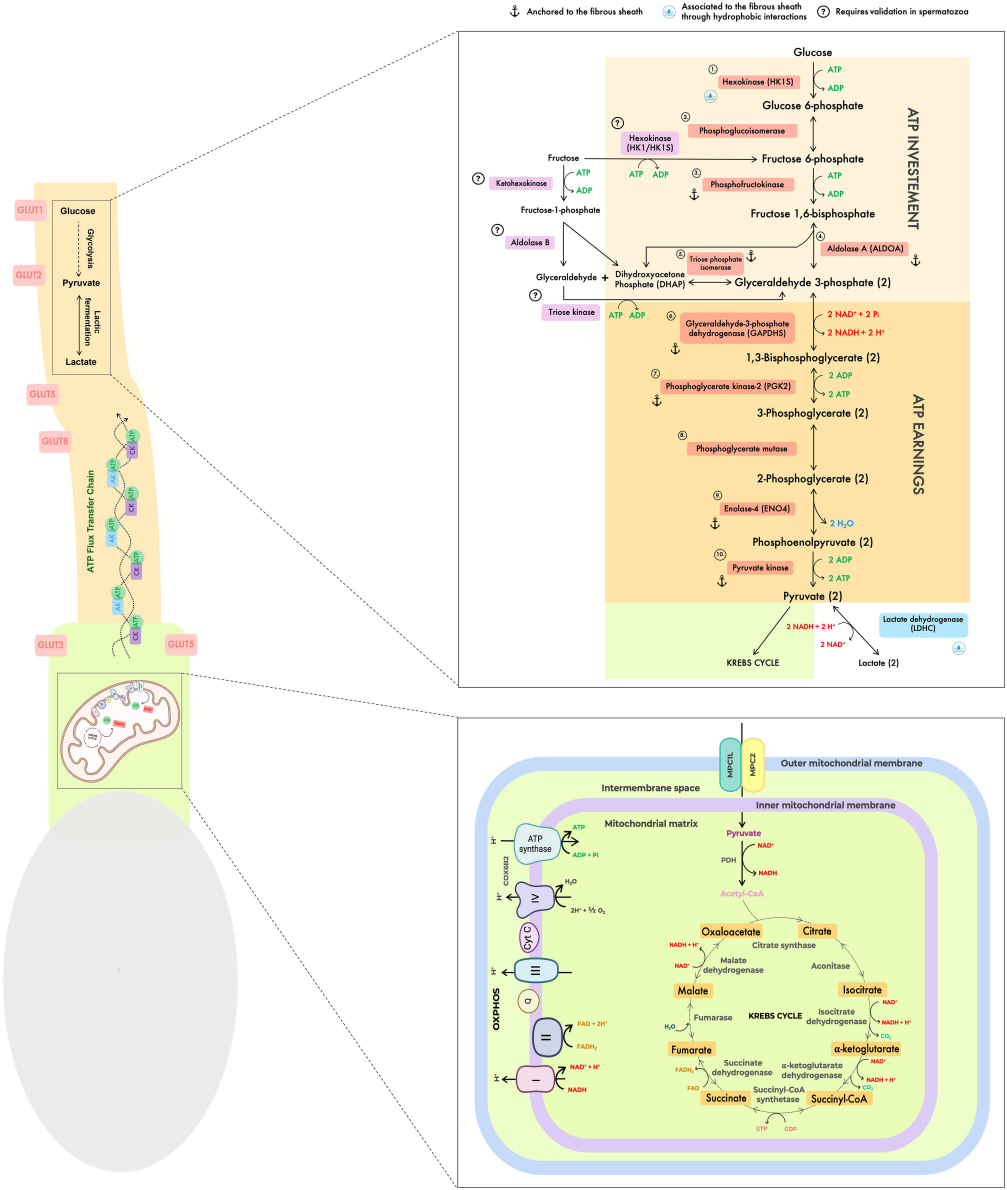
Schematic representation of the main metabolic pathways supporting human spermatozoa function. Glycolysis occurs predominantly in the principal piece of the flagellum and generates ATP locally, with glycolytic enzymes either tightly anchored to the fibrous sheath or associated through hydrophobic interactions. Besides glucose, fructose enters glycolysis through phosphorylation pathways leading to glycolytic intermediates. Pyruvate can either be oxidized to lactate, regenerating NAD^+^, or transported into the mitochondria and converted to acetyl-CoA, fueling the Krebs cycle to generate reducing equivalents (NADH and FADH_2_) that feed the electron transport chain (ETC) and oxidative phosphorylation (OXPHOS). Glucose uptake is mediated by distinct glucose transporters (GLUTs), strategically distributed along the spermatozoa. ATP produced in the midpiece is transferred to distal regions of the flagellum through phosphotransfer networks involving creatine kinase (CK) and adenylate kinase (AK), ensuring efficient energy delivery to support motility and hyperactivation. ADP, adenosine diphosphate; ATP, adenosine triphosphate; COX6B2, cytochrome c oxidase subunit 6B2; Cyt C, cytochrome C; FAD, flavin adenine dinucleotide; FADH_2_, reduced flavin adenine dinucleotide; GDT, guanosine diphosphate; GTP, guanosine triphosphate; MPC1L, mitochondrial pyruvate carrier 1-like; MPC2, mitochondrial pyruvate carrier 2; NAD^+^, oxidized nicotinamide adenine dinucleotide; NADH, reduced nicotinamide adenine dinucleotide; PDH, pyruvate dehydrogenase; Q, coenzyme Q10.

Glycolysis and OXPHOS are the two major metabolic pathways responsible for ATP production in human spermatozoa. As illustrated in **Figure 2**, these metabolic processes occur in a compartmentalized manner: OXPHOS occurs in mitochondria, located in the midpiece, while glycolysis occurs in the fibrous sheath of the principal piece, where glycolytic enzymes are located (8). Although OXPHOS is more efficient in terms of net energy production (30–32 ATP per oxidized glucose), this process is comparatively longer and dependent on oxygen availability; glycolysis is faster but less efficient in terms of net energy production (2 ATP per oxidized glucose) (19). Additionally, glycolysis has the added value of producing energy directly at the main beating part of the flagellum as opposed to OXPHOS, which occurs in the midpiece and from where the produced ATP requires transportation (20). This spatial separation led several authors to hypothesize that spermatozoa of most species, including humans, relied predominantly on glycolysis, despite OXPHOS being a significantly more efficient pathway to obtain ATP (21, 22); however, evidence suggests that ATP can be transported from the mitochondria to the flagellum via flux transfer chains (23, 24). Besides, the preferred energy pathway could be influenced by the capacitation state and different microenvironments found during spermatozoa’s journey throughout the female reproductive tract, including fluctuations in energy substrates and/or oxygen levels.

### 2.1 Glycolysis and ATP production in the flagellum

Glycolysis is a fundamental catabolic and cytoplasmic pathway that oxidizes one molecule of glucose to two molecules of pyruvate through a defined sequence of enzymatic steps, producing four ATP (net production of 2 ATP) and two reduced nicotinamide adenine dinucleotides (NADH) per molecule of glucose (19), as depicted in **Figure 2**. Glucose is a small polar monosaccharide that cannot pass through membranes without assistance, thus requiring facilitated transport by membrane-bound proteins. Membrane glucose-transporting proteins are divided into two main families: glucose-facilitating transporters (GLUTs, SLC2 family) and ATP-dependent sodium-coupled glucose transporters (SGLTs, SLC5 family). Using immunolocalization techniques, the presence of several GLUTs homologs have been extensively described in human spermatozoa: GLUT1 and GLUT2 were detected in the acrosomal region, principal and end pieces of the tail; GLUT3 exclusively in the midpiece; GLUT5 in the subequatorial region of the head, mid, and principal pieces; GLUT8 in the acrosome region and, at a lower intensity, in the post-acrosomal region and in the tail (25, 26). Conversely, there is no convincing evidence in the literature for SGLT family members in mature human spermatozoa, suggesting that these cells rely primarily on facilitative (i.e., GLUT-mediated) uptake rather than on sodium-coupled and ATP-dependent glucose transport.

Downstream of transport, human spermatozoa exhibit multiple germline-specific glycolytic isozymes that differ from their somatic counterparts in sequence, regulation, and subcellular organization (**Table 1**). Mechanistically, these differences are responsible for a compartmentalized glycolysis and, in some cases, for a distinct regulatory structure, tailored to the energetic needs of the flagellum where the dynein ATPases that confer motility are located (27). For example, a sperm-specific type 1 hexokinase (HK1S), the enzyme responsible for the irreversible phosphorylation of glucose into glucose 6-phosphate, which traps glucose in the cytoplasm, is found in the spermatozoa of humans and multiple other mammalian species (28, 29). Compared to the somatic counterpart, the N-terminal sequence lacks the mitochondrial porin binding domain, substituted by a germ cell-specific domain, thus suggesting that glycolysis and OXPHOS may be uncoupled in the male gamete (28). Unlike the other sperm-specific glycolytic enzymes, HK1S does not seem to exhibit an N-terminal extension that allows for fibrous sheath anchorage, although the germ cell-specific domain seems to bind to the fibrous sheath through hydrophobic interactions (30, 31). Sperm-specific glyceraldehyde 3-phosphate dehydrogenase (GAPDHS), the enzyme responsible for the reversible oxidative phosphorylation of glyceraldehyde 3-phosphate into 1,3-bisphosphoglycerate, shares only 70% of amino acid identity with somatic glyceraldehyde 3-phosphate dehydrogenase (GAPDH), containing a proline-rich N-terminal extension that plays a crucial role in anchoring to the fibrous sheath and substantial differences in the binding and catalytic activity of the allosteric site for oxidized nicotinamide adenine dinucleotide (NAD^+^) (32). Enolase-4 (ENO4) is a sperm-specific enolase found in human spermatozoa, responsible for the reversible dehydration of 2-phosphoglycerate into phosphoenolpyruvate (33, 34). Although the somatic enolase-1 (ENO1) is also present in human spermatozoa (35), evidence from mice suggests that ENO4 might be the principal enolase supporting the glycolytic activity in these cells. Besides showing that ENO4 also contains N-terminal extensions that contribute to the anchoring to the fibrous sheath compartment in mice spermatozoa, Nakamura et al. observed that *Eno4* knockout mice are infertile due to impaired spermatogenesis, reduced spermatozoa motility, and a high prevalence of coiled tails due to a disorganized fibrous sheath (36). Although these findings have not been demonstrated in humans, a recent study reported a case of homozygous *ENO4* mutation associated with asthenozoospermia and abnormal midpiece and flagellum morphology, resulting in infertility (37). Aldolase A (ALDOA), the enzyme responsible for the reversible conversion of fructose 1,6-bisphosphate into glyceraldehyde 3-phosphate and dihydroxyacetone phosphate, is the most abundant aldolase found in human spermatozoa (38). In mouse, three sperm-specific ALDOA isozymes were identified, two originated from retrotransposition (ALDOART1 and ALDOART2) and one by alternative splicing (ALDOA_V2), which also carried the N-terminal extensions that promote anchoring to the fibrous sheath (30, 39). Although human data are scarce, transcripts of *ALDOA_V2* were already detected in the human testis (40), whereas transcripts of *ALDOA-rs1*, a novel retroposed sequence, were identified in human spermatozoa (41). Although never validated in humans, phosphofructokinase (PFK), the enzyme that promotes the irreversible phosphorylation of fructose 6-phosphate to fructose 1,6-bisphosphate, and pyruvate kinase (PK), which catalyzes the transfer a phosphate group from phosphoenolpyruvate to ADP thereby producing ATP and pyruvate, were also reported to have sperm-specific structures in mammalian species that allow for fibrous sheath anchorage (30, 42–44).

**Table 1:** Molecular and catalytic properties, subcellular location, and putative role in capacitation and hyperactivation of glycolytic and lactic fermentation enzymes in human spermatozoa.

Key enzymes	Subcellular location	Source of evidence	Molecular and catalytic properties	Putative role in capacitation and hyperactivation	Functional validation
Hexokinase (HK)	HK1 is located in the acrosome region, midpiece and tail (184)	HK1 was validated by Western Blotting and Immunofluorescence (184)	Irreversible phosphorylation of glucose into glucose-6-phosphate *Evidence suggests that the mitochondrial porin-binding domain present in the N-terminal of the somatic isoform is substituted by a germ-cell-specific domain in the sperm-specific isoform, thought to bind to the fibrous sheath through hydrophobic interactions* (31)	Sustain the high ATP demand of capacitation and hyperactivation by controlling the first step of glycolysis	The indirect inhibition of HK using 2-DG, a competitive inhibitor of phosphoglucoisomerase, results in decreased motility and suppresses capacitation (69)
	HK2 was not validated in human spermatozoa	HK2 was identified in Proteomics data (185)			
	*A sperm-specific HK1 was identified in the tail and to a lesser degree in the midpiece and head of mouse spermatozoa* (186)	*Sperm-specific HK1 was validated by Immunofluorescence in mouse spermatozoa* (186)			
Phosphofructokinase (PFK)	PFKP (platelet-type isozyme) and PFKM (muscle-type isozyme) are located in the tail (187, 188)	Western Blotting and Immunofluorescence (187, 188)	Irreversible phosphorylation of fructose-6-phosphate to fructose-1,6-bisphosphate	Controls the rate-limiting step of glycolysis *PFK is hypothesized to stall glycolysis in seminal fluid due to the high concentrations of its allosteric inhibitors citrate and Zn^2+^* (19, 75–77, 82, 83)	No available data
	*A sperm-specific PFKM was identified in the tail of mouse spermatozoa* (42)	*Western Blotting and Immunofluorescence for sperm-specific PFKM in mouse spermatozoa* (42)	*Evidence suggests that the mouse sperm-specific PFKM is bound to the fibrous sheath* (42)		
Aldolase A (ALDOA)	Located in the equatorial and base region of the head, and in the tail (43, 189)	Western Blotting and Immunofluorescence (43, 189) *Transcripts for sperm-specific ALDOA were identified in mice and human spermatozoa* (30, 39, 41)	Reversible conversion of fructose-1,6-bisphosphate into glyceraldehyde-3-phosphate and dihydroxyacetone phosphate *Evidence suggests that ALDOA is bound to the fibrous sheath* (43) *Sperm-specific ALDOA carries the N-terminal extensions that promote anchoring to the fibrous sheath* (30, 39)	Supports capacitation by sustaining the high glycolytic flux required for hyperactivation *Evidence suggests that aldolase phosphorylation is an early event during capacitation, increasing its catalytic activity and thereby the glycolytic flux in mouse spermatozoa* (190)	No available data
Glyceraldehyde 3-phosphate dehydrogenase (GAPDH)	Somatic GAPDH was not identified in human spermatozoa *Sperm-specific GAPDH (GAPDHS) is located in the acrosome region and in the tail* (92, 191, 192)	GAPDHS was identified by Western Blotting and Immunofluorescence (92, 191, 192)	Reversible oxidative phosphorylation of glyceraldehyde 3-phosphate into 1,3-bisphosphoglycerate *GAPDHS contains a proline-rich N-terminal extension hypothesized to bind to the fibrous sheath* (43, 92) and a 3-fold higher catalytic efficiency compared to its somatic counterpart (193)	Supports capacitation by sustaining the high glycolytic flux required for hyperactivation *GAPDH is allosterically inhibited by Zn^2+^, suggesting that GAPDHS could stall glycolysis in the seminal fluid* (84)	The inhibition of GAPDHS using a specific inhibitor significantly decreased total motility and lactate production in human spermatozoa (32)
Phosphoglycerate kinase (PGK)	PGK1 and the sperm-specific PGK2 are both located in the tail (48)	Western Blotting and Immunofluorescence (48)	Reversible dephosphorylation of 1,3-bisphosphoglycerate into 3-phosphoglycerate with the production of ATP Evidence suggests that sperm-specific PGK2 is anchored to the fibrous sheath in human spermatozoa by binding to CABYR (52)	Evidence suggests that capacitation-induced phosphorylation and lactylation of PGK2 are associated with increased catalytic activity and glycolytic ATP production (45, 46) PGK2 interacts with CABYR, a calcium-binding and phosphorylation-regulated protein bound to the fibrous sheath of human spermatozoa, suggesting a crosstalk between glycolysis and capacitation signaling pathways (51, 52)	Inhibition of PKG2 using a specific anti-PGK2 antibody significantly decreased the penetrance capacity of human spermatozoa in a penetration assay (Kremer test). Inhibition of PGK1 using a specific anti-PGK1 antibody could not produce a similar effect (48)
Enolase (ENO)	ENO1 is located in the base of the head, midpiece and tail (35)	ENO1 was identified by Western Blotting and Immunofluorescence (35)	Reversible dehydration of 2-phosphoglycerate into phosphoenolpyruvate *Evidence suggests that ENO4 has N-terminal extensions that contribute to anchoring to the fibrous sheath in mouse spermatozoa* (36)	Supports capacitation by sustaining the high glycolytic flux required for hyperactivation Evidence suggests that ENO4 might be required to maintain the structural integrity of the sperm flagellum (36, 37)	Homozygous *ENO4* mutation was found in a case of asthenozoospermia with abnormal morphology in the midpiece and flagellum (37). No further functional data available *Eno4 knockout mice are infertile, exhibiting impaired spermatogenesis, sperm motility and a coiled tail with disorganized fibrous sheath* (36)
	ENO4 was identified in human spermatozoa, but its subcellular location has not been validated (33, 37)	ENO4 was identified using electrophoretic purification and activity evaluation (33, 194)			
	*ENO4 is located in the tail of mouse spermatozoa* (36)	*Immunofluorescence for ENO4 in mouse spermatozoa* (36)			
Pyruvate kinase (PK)	PKM (muscle isozyme) is located in the midpiece and tail (43)	Western Blotting and Immunofluorescence (43)	Irreversible transfer of a phosphate group from phosphoenolpyruvate to ADP, producing ATP and pyruvate *Evidence suggests that PK is bound to the fibrous sheath* (43)	Supports capacitation and hyperactivation by catalyzing the final ATP-producing step of glycolysis	No available data
Lactate dehydrogenase (LDH)	LDHC is mainly located in the post-acrosomal region of the head and to a smaller extent in acrosome region and in the principal piece (61) LDHA was not validated in human spermatozoa LDBH was identified in human spermatozoa, but its subcellular location has not been validated (59)	LDHC was identified by Western Blotting and Immunofluorescence (61, 195) LDHA was identified in proteomics data (38, 43) LDHB was identified by Western Blotting (59)	Reversible reduction of pyruvate into lactate. Evidence suggests that LDHC exhibits the highest affinity for pyruvate, catalytic activity and thermal stability among the isoforms, favoring the reduction of pyruvate into lactate (62) Proteomics data suggests that LDHA and LDHC could be bound to the fibrous sheath (43) *LDHA but not LDHC are bound to the fibrous sheath of mouse spermatozoa* (30)	Sustain a high rate of lactic fermentation to support the intense and localized glycolytic ATP production required for capacitation and hyperactivation	Exogenous pyruvate promotes capacitation and hyperactivation by increasing glycolytic ATP production. These effects were abolished by LDH inhibition with oxamate (46, 96)

Multiple glycolytic enzymes are the target of post-translational modifications (PTMs) during capacitation, as evidenced by several proteomics studies (45–47). However, few studies demonstrate that sperm-specific isoforms have different catalytic and/or regulatory properties as compared to the somatic counterparts, except for phosphoglycerate kinase-2 (PGK2). PGK2 is a sperm-specific isozyme of the somatic phosphoglycerate kinase-1 (PGK1), which catalyzes the first ATP-generating step of glycolysis through the reversible dephosphorylation of 1,3-bisphosphoglycerate into 3-phosphoglycerate (48, 49). PGK2 shares 88% of amino acid identity with PGK1 and is thought to have originated via retrotransposition of the X-linked *PGK1* gene to chromosome 6, compensating for *PGK1* silencing during meiotic sex chromosome inactivation that remains throughout the haploid period of spermatogenesis (49, 50). PGK2 is hypothesized to be central for human spermatozoa bioenergetics and motility, evidenced by the fact that the inhibition of PGK2, but not PGK1, significantly decreased the penetrance ability of human spermatozoa in the Kremer test (48). Moreover, decreased PGK2 expression was also implicated in ageing-dependent loss of fertility and asthenozoospermia cases (48). Although no differences had been noted when comparing PGK2 and PGK1 catalytic properties, PGK2 is distinguished by sperm-specific regulation and location: evidence suggests that a fraction of PGK2 is anchored to the fibrous sheath of the flagellum, further contributing to the spatial compartmentalization of glycolysis (48, 51). Herein, PGK2 is reported to interact with other testis-specific proteins, including the calcium-binding protein CPB86-IV (CABYR), a tyrosine phosphorylation-regulated protein that plays a role in the activation of the PKA signaling pathway, implying a crosstalk between energy metabolism and capacitation signaling pathways (51, 52). PGK2 is also the target of multiple PTMs, as capacitation-related residues phosphorylation and L-lysine lactylation have been associated with increased catalytic activity and ATP production, although the mechanistic details and site-specific effects are yet to be elucidated (45, 46). Based on these characteristics, PGK2 has been considered a potential target for male contraceptive development (53).

A high glycolytic flux is typically accompanied by a high flux of lactic fermentation. In fact, some authors argue that the anaerobic conversion of pyruvate, the traditional glycolytic endpoint, into lactate is the actual physiological endpoint of glycolysis (54, 55). The reversible reduction of pyruvate into lactate, catalyzed by the enzyme lactate dehydrogenase (LDH), regenerates cytosolic NAD^+^ from NADH and thereby sustains the glycolytic turnover (56). Curiously, the sperm-specific lactate dehydrogenase (LDHC) was the first germ cell-specific isozyme to be described in human spermatozoa (57, 58). Although the somatic LDH subunit LDHB was already identified and validated (59), and proteomics data suggests that LDHA is also present in human spermatozoa (38, 43), LDHC appears to be the predominant isozyme present in mammalian spermatozoa, with quantitative evidence in mouse (>80% of total activity (60)) and qualitative/electrophoretic evidence in humans (57, 58). Spatially, LDHC is predominantly present in the post-acrosomal region of the head and, to a smaller extent, in the acrosome region and principal piece of human spermatozoa (61). Although validation is required, proteomics data suggests that LDHC and LDHA might be bound to the fibrous sheath, similarly to other glycolytic enzymes (43). Biochemically, LDHC shows distinct properties as compared to LDHA (75.3% amino acid identity) and LDHB (69.8% amino acid identity), characterized by a higher affinity for pyruvate, greater heat stability, and a predominant catalytic activity towards lactate and NAD^+^ production (62). These features are well-suited to sustain a high rate of lactic fermentation and thereby support the intense, localized glycolytic ATP production required for hyperactivated motility. Corroborating the importance of LDHC for male fertility, reduced LDHC expression and activity are correlated with impaired progressive motility in clinical cohorts, leading LDHC (as well as the sperm-specific glycolytic enzymes GAPDHS and PGK2) to be proposed as a potential biomarker for immaturity-related and asthenozoospermia phenotypes (63). Due to its germ cell-specificity and critical role in human spermatozoa bioenergetics, LDHC was also proposed as an attractive target for male contraception (64).

Glucose is the *de facto* energy substrate that fuels glycolysis. However, at the beginning of the mature spermatozoa’s journey, glucose is not readily available and therefore not the principal energy substrate. The human seminal fluid contains low levels of glucose (0.2-0.4 mM) but high levels of fructose (5–30 mM) (65–68). Fundamentally, fructose is the principal energy substrate in the seminal fluid, whereas fructolysis shares most of the enzymes with glycolysis and produces the same net energy per oxidized molecule. As the metabolic pathway is mostly shared, it is not surprising that fructose can support motility and hyperactivation as efficiently as glucose in *in vitro* conditions, although producing a slightly decreased response to calcium ionophore plus pentoxifylline-induced acrosome reaction (69). Supporting this evidence, some studies highlighted that low fructose levels in the seminal fluid are often associated with asthenozoospermia (70, 71). Fructose uptake occurs via fructose transporter GLUT5 and GLUT2, both located in the head and principal piece in human spermatozoa (25, 72). However, the enzymes responsible for fructolysis where never identified or functionally validated in human spermatozoa. If following the canonical pathway, fructolysis would begin in the cytoplasm with ketohexokinase (KHK; also known as hepatic fructokinase), which phosphorylates fructose to fructose-1-phosphate and thereby traps fructose inside the cell. As shown in **Figure 2**, fructose-1-phosphate would then require cleavage into glyceraldehyde and dihydroxyacetone phosphate (DHAP) by aldolase B; glyceraldehyde would then be phosphorylated by triose kinase to glyceraldehyde-3-phosphate, while DHAP would be converted into glyceraldehyde-3-phosphate by triose-phosphate isomerase, allowing both intermediates to enter glycolysis. Alternatively, at high levels, fructose may be phosphorylated by hexokinase (HK1 or HK1S) to fructose-6-phosphate, which enters glycolysis directly (73). Despite the extensive evidence that human spermatozoa oxidize fructose, the enzymes KHK and aldolase B have never been formally identified in these cells, nor has fructose utilization by HK1/HK1S been experimentally validated. Some authors suggest that KHK would be the likely responsible for fructose oxidation, as this pathway would have the advantage of bypassing PFK, a finely regulated rate-limiting step of the glycolytic pathway (74). PFK is highly sensitive to allosteric regulation, being activated by fructose-2,6-bisphosphate and a low ATP/adenosine monophosphate (AMP) ratio while being inhibited by citrate and Zn^2+^ (19), two factors that are highly present in the seminal fluid. In fact, citrate levels in the seminal fluid (5–50 mM (75–77)) are up to 1000 times higher than the reported K_i_ range for PFK (0.05-0.75 mM (78, 79)); whereas Zn^2+^, a known decapacitating factor, is reported at levels (typically between 1.53-3.06 mM (80), with some studies reporting levels up to 6.55 mM (81)) up to 4333 times higher than the reported K_i_ range for PFK (1.5-230 µM (82, 83)), suggesting that both factors can induce a strong but reversible inhibitory effect while spermatozoa are in the seminal fluid. Besides, Zn^2+^ is a strong inhibitor of GAPDH (reported K_i_ range of 77-111 µM (84)), suggesting a potential inhibitory effect on glycolysis if a similar effect occurs in GAPDHS; however, further studies are needed to confirm this hypothesis. Overall, the conditions found in the seminal fluid, which are transient in nature, seem to maintain a balance between vitality, motility, and a quiescent metabolic state, preventing premature capacitation and acrosome reaction due to the presence of decapacitating factors.

Later in the spermatozoa’s journey through the female reproductive tract, and in contrast with the seminal fluid, uterine and oviductal secretions contain very low levels of fructose but do contain glucose, pyruvate, and lactate in varying proportions depending on the fluid and, according to some studies, the stage of the female reproductive cycle. Weed and Carrera reported that glucose levels in the cervical mucus peak at ovulation, averaging 8.3 mM, and that higher glucose content was positively associated with spermatozoa motility and vitality, concluding that optimal conditions occur at approximately 11 mM glucose (85). Hugh et al. reported a similar pattern in endometrial secretions, with glucose rising from approximately 2.8 mM to 6.7 mM during ovulation (86). However, the data found in the literature are not consistent. Casslén and Nilsson reported that uterine glucose levels are similar to blood serum (5.1-5.7 mM) with no cycle-dependent changes (87). Likewise, Gardner et al. observed stable uterine glucose levels (3.15 ± 0.31 mM) across the cycle (88). Conversely, the authors noted a midcycle decrease in oviductal glucose (14-16^th^ day, 0.50 ± 0.21 mM) as compared to the follicular phase (3.11 ± 0.64 mM). This decrease was associated with increased lactate levels during midcycle (10.50 ± 1.48 mM *vs* 4.87 ± 0.63 mM in the follicular phase), prompting the authors to suggest that reduced glucose may prepare the oviductal microenvironment for early embryo development since glucose may inhibit embryogenesis (88). Nevertheless, this extensive decrease in oviductal glucose levels at midcycle is challenged in other studies. Lippes et al. observed that glucose levels remain constant in the human oviduct fluid after ovulation (3.04 ± 0.83 mM pre-ovulation *vs* 2.40 ± 0.51 mM post-ovulation) (89). Similarly, Utsunomiya et al. reported an average glucose of 3.4 mM at midcycle (14-16^th^ day), denoting that glucose is the principal energy substrate available in the oviductal fluid at that stage (90). Although absolute quantification is difficult because sampling is invasive and volumes are small, which undermines data validity, all but one study identified glucose as the principal energy substrate in uterine and oviductal fluids. These findings accentuate the importance of glucose as the main energy substrate for human spermatozoa bioenergetics within these microenvironments, where capacitation and hyperactivation take place.

The literature is consensual on the view that glycolysis is required for human spermatozoa capacitation and hyperactivation (8, 20, 22, 91). Much of this conclusion rests on the findings by William and Ford, who demonstrated that glucose and fructose are efficient inducers of *in vitro* capacitation of human spermatozoa (69). These authors reported that glucose and fructose equally increased the percentage of hyperactivated spermatozoa, which was also associated with increased motility and ATP/adenosine diphosphate (ADP) ratio. The effects of glucose were abolished in the presence of 2-deoxyglucose (2-DG), a competitive inhibitor of phosphoglucoisomerase, further evidencing that glycolysis is required for human spermatozoa capacitation (69). Posterior studies reinforced this conclusion, as the selective inhibition of GAPDHS led to glycolysis inhibition and decreased motility (32); whereas the oxidative inactivation of GAPDHS by reactive oxygen species (ROS) similarly suppresses the glycolytic activity and motility in human spermatozoa (92). Interestingly, human spermatozoa tolerate high glucose levels without functional loss. Portela et al. demonstrated that high glucose levels (25–50 mM) are as effective as physiological glucose levels (5.5 mM) in inducing the capacitation of human spermatozoa, as well as maintaining their motility and vitality for up to 48h (93). Supporting these findings, Carrageta et al. showed that high glucose levels (11–22 mM) produce a similar effect as physiological glucose levels (5.5 mM) in inducing the protein tyrosine residues phosphorylation cascade and increasing total motility in human spermatozoa, an effect that was omitted in the absence of glucose (94). Overall, these data indicate that, within a wide range of concentrations, glucose excels in the support of the metabolic demands of capacitation. Mechanistically, capacitation and glycolysis appear to reinforce one another in a positive cycle: glycolysis is required for capacitation and hyperactivation, while capacitation signaling increases the glycolytic flux to meet the heightened energetic demands of hyperactivated motility (94, 95). The reliance of human spermatozoa on lactic fermentation further evidences the dependence on glycolysis. Hereng et al. showed that pyruvate supplementation raised intracellular ATP levels, progressive motility, protein tyrosine residues phosphorylation, and hyperactivation in human spermatozoa as compared to glucose alone (96). These effects were associated with an increased NAD^+^ regeneration through LDH activity, which increased the glycolytic flux. In turn, the absence of effects when oxamate, a known LDH competitive inhibitor, was present corroborates these findings (96). Similarly, Yan et al. reported that oxamate markedly reduced progressive motility, concomitant with lower ATP production due to slowed glycolytic flux (46). In sum, multiple complementary lines of evidence support a model in which glycolysis is both necessary for, and upregulated by, capacitation in human spermatozoa, being required to sustain the high ATP demand of hyperactivation.

Besides lactic fermentation, pyruvate is also the main mitochondrial energy substrate, with expression of the canonical mitochondrial pyruvate carrier 1 and 2 (MPC1/MPC2) subunits in the midpiece (97). In addition, human spermatozoa also express a sperm-specific paralogue, the mitochondrial pyruvate carrier 1-like (MPC1L), which is functionally equivalent to MPC1 (98). Inside the mitochondria matrix, pyruvate can be converted into acetyl-CoA by pyruvate dehydrogenase (PDH). Similar to the glycolytic enzymes, human spermatozoa express a sperm-specific E1α subunit of the PDH complex (PDHA2). PDHA2 shares an 87% amino acid identity with the somatic chromosome X-linked PDHA1 paralogue and, as in the case of PGK2, it compensates for PDHA1 silencing during meiotic sex chromosome inactivation that remains throughout the haploid period of spermatogenesis (99). Interestingly, PDHA2 shares the same catalytic properties but is less efficiently phosphorylated/dephosphorylated by pyruvate dehydrogenase kinase (PDK) and pyruvate dehydrogenase phosphatase (PDP), respectively, than the somatic PDHA1 (100). Acetyl-CoA then condensates with oxaloacetate to originate citrate, catalyzed by citrate synthase, within the first step of the Krebs cycle (also known as the tricarboxylic cycle - TCA). The Krebs cycle is the main source of the reducing equivalents, producing two molecules of NADH and one molecule of reduced flavin adenine dinucleotide (FADH_2_) per cycle. These reducing equivalents, in turn, fuel the electron transfer chain (ETC) to promote OXPHOS.

### 2.2 Oxidative phosphorylation and ATP production in the midpiece

In spermatozoa, the mitochondria are concentrated in the midpiece, wrapped helically around the flagellum to form the thick mitochondrial sheath. Although spermatozoa have significantly fewer mitochondria as compared to the typical somatic cell (70–80 in the midpiece (101)), these organelles are central in spermatozoa bioenergetics as they host a set of metabolic processes: the Krebs cycle, fatty acids β-oxidation, amino acid metabolism and OXPHOS. The OXPHOS pathway, summarized in **Figure 2**, occurs in the inner mitochondrial membrane through the action of the ETC, which consists of four multi-subunit protein complexes (I-IV) and two mobile electron carrier molecules (ubiquinone and cytochrome c), and ATP synthase (complex V) (19). Electrons derived from NADH and FADH_2_ enter the ETC (NADH at complex I, FADH_2_ at complex II) and are passed sequentially through the complexes to molecular oxygen, the final electron acceptor, which is reduced to H_2_O at complex IV. As electrons flow, complexes I, III and IV translocate protons from the mitochondrial matrix into the intermembrane space, generating an electrochemical gradient. In turn, the return flow of protons through ATP synthase drives phosphorylation of ADP into ATP. Beginning in glycolysis and ending in the Krebs cycle and OXPHOS, the complete oxidation of one molecule of glucose generates 30–32 molecules of ATP (19).

Compared to glycolysis, OXPHOS yields far more ATP per oxidized substrate despite requiring oxygen, whereas glycolysis is kinetically faster and, due to the flagellum-anchored glycolytic enzymes, can deliver ATP directly at the site of dynein activity. Although the centrality of glycolysis for human spermatozoa capacitation and hyperactivation is widely accepted, the relative contribution of OXPHOS to these processes has been debated for decades (**Table 2**) (20, 21). It is widely accepted that healthy and active mitochondria are associated with spermatozoa quality: mitochondrial membrane potential correlates positively with vitality and progressive motility in human spermatozoa (102, 103), and mitochondrial respiratory control ratio (a method used to estimate how tightly oxygen consumption is coupled with ATP production) is positively associated with progressive motility (104). Supporting these findings, a decreased activity of complexes I, II and IV is often reported in asthenozoospermia cases (105). Interestingly, spermatozoa possess a sperm-specific cytochrome paralogue of the complex IV subunit VIb, the cytochrome c 6B2 subunit oxidase (COX6B2) (106). Functional studies show that COX6B2 has enhanced activity as compared to the somatic isoform, increasing OXPHOS flux and NAD^+^ regeneration (107). Abramczyk et al. also observed that motile human spermatozoa predominantly contained cytochrome c in its oxidized form, constituent with increased OXPHOS flux, whereas a predominant reduced form was associated with impaired ETC and reduced ATP production (108). Counterarguments against a major role of OXPHOS in capacitation and hyperactivation remain. Specifically, the potential low oxygen availability in the female reproductive tract and the logistical challenge of supplying ATP from the mitochondria to the distal flagellum have led some authors to argue against a dominant role of OXPHOS in physiological contexts (20, 21). Furthermore, the wide inter-species and/or methodological differences often complicate extrapolation to human physiological conditions.

**Table 2:** Molecular and catalytic properties, subcellular location, and putative role in capacitation and hyperactivation of Krebs cycle and oxidative phosphorylation (OXPHOS) enzymes in human spermatozoa.

Key enzymes	Subcellular location	Source of evidence	Molecular and catalytic properties	Putative role in capacitation and hyperactivation	Functional validation
Pyruvate dehydrogenase complex (PDH)	Sperm-specific E1α subunit (PDHA2) and E1β subunit (PDHB) are located in the posterior region of the head (196) Proteomics data suggests that PDHA2, somatic E1α subunit (PDHA1), and PDHB are also located in the tail (125)	Western blotting and Immunofluorescence with PDHA antibody with potential cross-reactivity with PDHA1/2 and PDHB (196) PDH1, PDH2 and PDHB were identified in a proteomics study (125)	Irreversible oxidative decarboxylation of pyruvate to acetyl-CoA PDHA2 is the sperm-specific E1α isoform encoded by the *PDHA2* gene on chromosome 4. It replaces X-linked *PDH1A* during and post-meiosis, maintaining PDH activity. Although catalytically similar, PDHA2 shows altered phosphorylation regulation (100) *In contrast to somatic-PDH, sperm-PDH activity is regulated by L-malate in mouse, rat, and rabbit* (197)	Supports capacitation and hyperactivation by fueling the Krebs cycle, promoting ATP, reducing equivalents, and ROS production to sustain the high energy demands and the activation of cAMP/PKA signaling *Evidence suggests that PDH exhibits tyrosine phosphorylation during capacitation, and its activity positively correlates with hyperactivation in hamster spermatozoa* (196)	No available data
Citrate synthase (CS)	CS has been identified in human spermatozoa head and tail proteomics, but antibody validation is lacking (125, 198) *CS is localized in the midpiece and tail of mouse spermatozoa* (199)	CS was identified in proteomics studies (125, 198) *CS and eCS were validated by Western blotting and Immunofluorescence in mice* (199)	Irreversible condensation of acetyl-CoA with oxaloacetate to produce citrate *eCS is a sperm-specific isoform encoded by the Csl gene in mice that lacks the mitochondrial targeting sequence. Evidence suggests it maintains CS activity* (199)	CS is a rate-limiting enzyme of the Krebs cycle Evidence suggests citrate increases nitric oxide production during human spermatozoa capacitation (200) *Evidence suggests eCS regulates acrosome reaction in mice via cAMP signaling* (201)	CS activity is used as an index of mitochondrial mass and function in human spermatozoa (105). No further functional data available *eCS-deficient mouse spermatozoa fail to undergo acrosome reaction (*201*).*
	*Sperm-specific extra-mitochondrial form of CS (eCS) was identified in the head, tail and midpiece of mouse spermatozoa* (199)				
Aconitase (ACO2)	ACO2 is located in the midpiece (202)	Western Blotting and Immunofluorescence (202)	Reversible isomerization of citrate to isocitrate	Evidence suggests that ACO2 may have a role in sperm motility (202)	Reduced ACO2 protein levels were identified in asthenozoospermia, and sperm motility was rescued by isocitrate (202). No further functional data available
Isocitrate dehydrogenase (IDH)	IDH was not validated in human spermatozoa. Proteomics evidence suggests its presence in the tail (125) *Cytosolic NADP^+^-dependent IDH was identified in the tail of porcine spermatozoa* (203)	IDH1, IDH2, IDH3A, IDH3B, and IDH3G were identified in proteomics studies (125, 180)	Irreversible oxidative decarboxylation of isocitrate to α-ketoglutarate, generating NADH and CO_2_	*Evidence from porcine spermatozoa suggests that a decrease in the cytosolic NADP^+^-dependent IDH activity is involved in the increased ROS production that induces capacitation and hyperactivation* (203)	No available data in human spermatozoa *Inhibition of IDH prevented capacitation and acrosome reaction in boar spermatozoa* (204)
α-ketoglutarate dehydrogenase	α-ketoglutarate dehydrogenase was not validated in spermatozoa. Proteomics evidence suggests its presence in the tail (125)	Proteomics study (125)	Catalyzes the oxidative decarboxylation of α-ketoglutarate to succinyl-CoA	Likely supports capacitation by sustaining mitochondrial oxidative metabolism	No available data
Succinyl-CoA synthetase	Succinyl-CoA synthetase was not validated in human spermatozoa. Proteomics evidence suggests its presence in the tail (125)	Proteomics study (125)	Reversible conversion of succinyl-CoA to succinate, generating GTP via substrate-level phosphorylation	Succinyl-CoA synthetase is a key enzyme of the Krebs cycle and supports mitochondrial ATP/GTP production via substrate-level phosphorylation, contributing to the energy supply required for capacitation and hyperactivation	No available data
Succinate dehydrogenase (SDH or Complex II)	SDH subunit D (SDHD) is mainly located in the midpiece and to a lesser extent in the head (205)	Immunofluorescence (205)	Reversible oxidation of succinate into fumarate while producing FADH_2_	Likely supports capacitation and hyperactivation by linking the Krebs cycle to the ETC, contributing to mitochondrial ATP production and redox balance	SDH activity correlates with sperm motility (105). No further functional data available
NADH:ubiquinone oxidoreductase (Complex I)	NADH:ubiquinone 1 alpha subcomplex, 13 (NDUFA13) is mainly located in the midpiece, with occasional signals detected in the head (206)	NDUFA13 was identified by Western blotting and Immunofluorescence (206)	Catalyzes the oxidation of NADH to NAD^+^ while simultaneously reducing ubiquinone, coupled to proton translocation across the mitochondrial inner membrane	Supports the high energy demands of spermatozoa motility	Complex I inhibition causes a decline in ATP levels and progressive motility (96)
Ubiquinol:cytochrome C oxidoreductase (Complex III)	Proteomics data suggest the presence of cytochrome b-c1 complex subunits 1 (UQCRC1), 2 (UQCRC2), 7 (UQCRB), 8 (UQCRQ), and Rieske (UQCRFS1), and cytochrome c1 (CYC1) in the tail, but antibody validation is lacking (125)	Proteomics study (125)	Catalyzes the oxidation of ubiquinol (QH_2_) and the reduction of cytochrome c, transferring electrons through the Q-cycle, coupled to proton translocation across the mitochondrial inner membrane	Contributes to mitochondrial ATP and ROS production (104, 207)	Complex III inhibition causes a decline in ATP levels (104)
Cytochrome c oxidase (Complex IV)	Sperm-specific Cytochrome c 6B2 subunit oxidase (COX6B2) is located in the midpiece (208)	Immunofluorescence (208)	Catalyzes the transfer of electrons from cytochrome c to O_2,_ producing H_2_O, coupled to proton translocation across the mitochondrial inner membrane Evidence suggests that the sperm-specific COX6B2 paralogue has enhanced activity compared to the somatic complex IV subunit Vib isoform, increasing OXPHOS flux and NAD^+^ regeneration (107)	Supports mitochondrial oxidative phosphorylation and mitochondrial ATP production	Reduced COX6B2 abundance and mislocalization in asthenozoospermia suggest impaired translocation and compromised OXPHOS activity (208) Complex IV inhibition impaired progressive motility, capacitation-associated tyrosine phosphorylation, and hyperactivation (96)
ATP synthase (Complex V)	ATP-synthase β subunit (ATP5B) is located in the midpiece (209)	Western blotting and Immunofluorescence (209)	Catalyzes the synthesis of ATP from ADP and phosphate in the mitochondria	Supports mitochondrial oxidative phosphorylation and mitochondrial ATP production	ATP synthase inhibition with oligomycin causes a decline in ATP concentration (210)

As molecular oxygen is the terminal electron acceptor in the ETC, OXPHOS is fundamentally an aerobic pathway whose capacity relies on the microenvironment’s oxygen availability. In many cell types, OXPHOS can operate at extremely low oxygen levels and becomes severely impaired only if oxygen partial pressure (pO_2_) falls under 1 mmHg (109, 110). Such extreme hypoxic conditions are unlikely to be encountered by human spermatozoa during their journey throughout the female reproductive tract. Although the vaginal canal exhibits low oxygen levels [basal pO_2_ is around 3.8 ± 0.9 mmHg (111)], molecular oxygen availability progressively increases as spermatozoa ascend through the uterus (average 13–42 mmHg) and into the oviducts (average 53–60 mmHg) (112). Additionally, some studies highlight that pO_2_ is influenced by the menstrual cycle. For instance, Yedwab et al. recorded an increase in pO_2_ in the uterus by 86-90% at the ovulatory phase in humans (113). These oscillations in pO_2_ in the uterus seem to occur due to the action of estrogen and progesterone, as the peaks of pO_2_ were recorded under low estrogen and high progesterone levels (112, 114). Taken together, these observations indicate that oxygen availability is not generally limiting mitochondrial respiration in the peri-fertilization window, dethroning the argument of low oxygen availability.

The claim that ATP is transported from mitochondria to the distal flagellum via a dedicated energy flux transfer chain is currently difficult to support, largely due to the scarcity of strong evidence in human spermatozoa. Energy flux transfer chains describe a mechanism where a cascade of enzymatic reactions relays energy-rich molecules (such as phosphoryls) from one enzyme to the next until they reach the site of consumption, allowing rapid and localized regeneration of ATP where it is required (115). Theoretically, what would drive phosphoryl’s motion through an enzymatic chain would be the changes in concentration of specific substrates. In this case, lower concentrations of ATP in the principal piece of the flagellum compared to the midpiece would generate a “wavefront” that propagates phosphoryl transfer along the enzymatic chain in a domino-like manner (115). Peter Mitchell also proposed that the principle of vectorial ligand conduction could present itself as an essential player for the flux in the enzymatic chain (116). In this principle, certain enzymes, such as creatine kinase (CK) or adenylate kinase (AK), would incorporate the chain and facilitate the transfer of the phosphoryl groups between ATP-consuming and ATP-generating sites (116). Although these mechanisms are well described in non-mammalian spermatozoa, where clear spatial separation of energy production and consumption is bridged by effective phosphotransfer systems (117, 118), the evidence in mammals, and particularly in humans, is much more limited (**Table 3**). Creatine kinase (CK, also known as creatine phosphokinase or phosphocreatine kinase) catalyzes the reversible conversion of creatine and ATP into phosphocreatine and ADP, enabling phosphocreatine to be used as an energy reservoir for rapid energy buffering and for intracellular energy transport through the phosphocreatine shuttle (119). The canonical model postulates that a mitochondrial CK, present in the intermembrane space, converts mitochondrial ATP into phosphocreatine using creatine imported from the cytosol. In turn, a cytosolic CK reconverts phosphocreatine into ATP at sites of consumption, thereby acting as an ATP shuttle between the mitochondria and cytosol (119). Four isoforms have been identified in mammals: the cytosolic homodimer muscle-type CK (CK-M), homodimer brain-type CK (CK-B), the hybrid heterodimer CK-MB, and the mitochondrial CK (CK-Mi) (120). In human spermatozoa, the isoforms CK-B, CK-MB and CK-Mi have been identified (120, 121), although its functional validation remains to be elucidated. Functionally relevant evidence is stronger in animal models, for example, Umehara et al. showed that creatine, which is found at high levels in follicular and oviductal fluids after ovulation, increases ATP production, enhances capacitation, and improves *in vitro* fertilization success rates in mice (122). In humans, early *in vitro* studies indicate that creatine or phosphocreatine improves spermatozoa motility and velocity (123), but mechanistic studies on the effects of creatine on human spermatozoa capacitation and hyperactivation are inexistent. An approximation towards a mechanistic explanation was obtained by Yeung et al., who showed that the inhibition of CK causes a great decrease in human spermatozoa total motility and intracellular ATP levels if lactate is the only energy substrate in the media (23). When glucose was available, CK inhibition did not change total motility but did impair curvilinear velocity, straight-line velocity and linearity, suggesting that CK acts as a supplementary mechanism that supports fine-tuned aspects of flagellar motility (23).

**Table 3:** Molecular and catalytic properties, subcellular location, and putative role in capacitation and hyperactivation of ATP flux transfer chain enzymes in human spermatozoa.

Key enzymes	Subcellular location	Source of evidence	Molecular and catalytic properties	Putative role in capacitation and hyperactivation	Functional validation
Creatine Kinase (CK)	CK-Mi (mitochondrial), CK-B (brain-type) and heterodimer-type CK-MB (muscle-brain type) were identified in human spermatozoa, but its subcellular location has not been validated (120, 121)	CK-Mi, CK-B, and CK-MB, were identified by Electrophoretic separation and activity evaluation (120) CK-Mi and CK-B were identified by Western Blotting (121)	Reversible conversion of creatine and ATP into phosphocreatine and ADP (phosphocreatine shuttle)	It is hypothesized to support motility by shuttling high-energy phosphoryl groups from the mitochondria to the distal flagellum via phosphocreatine, enabling rapid energy buffering and intracellular energy transport	Creatine and phosphocreatine improves in vitro motility and velocity of human spermatozoa (123) CK inhibition by DNFB reduced total motility and intracellular ATP levels under lactate-only conditions. In the presence of glucose, CK inhibition resulted in impaired motility pattern, denoted by a reduced curvilinear velocity, straight-line velocity, and linearity (23)
Adenylate kinase 1 (AK1)	AK1 is located in the tail (127)	Western Blotting and Immunofluorescence (127)	Reversible conversion of ATP and AMP into two ADP molecules	Facilitate the transfer of the phosphoryl groups between ATP-producing and ATP-consuming sites Regulation of OPXHOS flux by controlling mitochondrial ADP availability	AK1 was found downregulated in the tail of spermatozoa of men with severe oligoasthenoteratozoospermia (127). No further functional data is available
Adenylate kinase 2 (AK2)	Proteomics data suggests that AK2 is located in the tail (125) *AK2 is located in the mitochondrial sheath of mouse spermatozoa* (211, 212)	Proteomics data (125, 126) *Western Blotting, Immunofluorescence and Immunogold labelling (Electronic Microscopy) in mouse spermatozoa* (211, 212)			No available data
Adenylate kinase 4 (AK4)	Evidence suggests that AK4 is present in human spermatozoa, but its subcellular location was never validated (126)	Proteomics data (126)			No available data
Adenylate kinase 6 (AK6)	Evidence suggests that AK6 is present in human spermatozoa, but its subcellular location was never validated (126)	Proteomics study (126)			No available data
Adenylate kinase 7 (AK7)	AK7 is located in the tail (128, 129)	Western blotting and Immunofluorescence (128, 129)			Two homozygous missense mutations were identified to produce a sperm-specific loss of AK7, which were associated with multiple morphological abnormalities in the tail, low motility, and total fertilization failure in ICSI cycles (128, 129)
Adenylate kinase 8 (AK8)	AK8 is present in human spermatozoa, but its cellular location was never validated (24) *AK8 is located in the tail of mouse spermatozoa* (24)	Western blotting (24)			An AK8 variant was associated with decreased AK8 protein levels in spermatozoa of a man with severe asthenozoospermia (24) *Ak8 knockout mice produced spermatozoa with no motility and mitochondrial ATP accumulation, suggesting an impaired ATP flux chain from the mitochondria to the principal piece* (24)
Adenylate kinase 9 (AK9)	AK9 is located in the tail (24)	Western blotting and Immunofluorescence (24)			Two AK9 variants were associated with absent AK9 protein in the tail of spermatozoa of men with severe asthenozoospermia (24) *Ak9 knockout mice produced spermatozoa with no motility and mitochondrial ATP accumulation, suggesting impaired ATP flux chain from the mitochondria to the principal piece* (24)

Adenylate kinase (AK, also known as myokinase) catalyzes the interconversion of ATP and AMP into two ADP molecules, controlling OXPHOS flux by increasing ADP availability in the mitochondria (124). Nine isozymes (AK1-9) have been identified in mammals, showing distinct subcellular locations: AK1, AK5, AK7 and AK8 are predominantly cytosolic; AK2 resides in the mitochondrial intermembrane space; AK3 and AK4 are located in the mitochondrial matrix; and AK6 and AK9 are mainly located in the nucleus (124). Although the mechanisms are not fully understood, recent genetic and clinical evidence support an AK-dependent phosphotransfer pathway in human spermatozoa. Among the known isozymes, only AK3 and AK5 have never been identified in human spermatozoa. While AK2, AK4, and AK6 have been detected in proteomic datasets and still require orthogonal validation (125, 126), available evidence suggests that AK1, AK7, AK8, and AK9 might contribute to motility and fertilizing ability of human spermatozoa. For instance, Liang et al. showed that AK1 was downregulated in the tail of spermatozoa of men with severe oligoasthenoteratozoospermia as compared to men with normozoospermia (127). Likewise, Lorès et al. identified AK7 in the tail of human spermatozoa (128). In this study, these authors identified a homozygous missense mutation in the *AK7* gene (c.2018T > C; p.Leu673Pro) which resulted in the loss of AK7 protein exclusively in spermatozoa, producing cells with multiple morphological abnormalities in the tail and severe asthenozoospermia (128). Supporting these findings, Xiang et al. found another homozygous missense mutation in the *AK7* gene (c.1846G > A; p.E616K) associated with asthenozoospermia and total fertilization failure in two ICSI cycles (129). More recently, Wu et al. found reduced AK8 and AK9 protein levels in two cases of severe asthenozoospermia (24). Using *Ak9* and *Ak8* knockout mice, followed by validation in the AK9-deficient human spermatozoa, these authors showed that ATP accumulates in mitochondria rather than being made available to the flagellum in genetically altered cells, indicating that AK9 (and potentially AK8) regulates ATP transfer in the axoneme of human spermatozoa (24). Complementing these findings, O’Challaghan et al. found that *Ak9* knockout mice produce immotile spermatozoa with low ATP levels and an inability to hyperactivate or penetrate the *zona pellucida* (130), although these effects remain to be demonstrated in humans. To summarize, flux transfer chains, such as CK or AK-mediated flux chains, exploit an enzymatic cascade to shuttle high-energy phosphoryl groups from regions of ATP production, such as the mitochondria, to sites of ATP consumption, offsetting the continuous hydrolysis of ATP to ADP that fuels spermatozoa motility. While current findings strongly implicate CK and AK-mediated flux chains in spermatozoa bioenergetics and motility regulation, their specific roles in human spermatozoa capacitation and hyperactivation require further research.

Finally, inter-species differences in bioenergetics and methodological variability must not be overlooked when extrapolating to human physiology. For instance, while spermatozoa from rodents seem to rely more on glycolysis (131), spermatozoa from stallions (132), bulls (133), goats (134), and pigs (14) depend more heavily on OXPHOS. The great differences between species render the comparison with humans highly challenging, and even the rodents, which are widely used as models in health sciences, exhibit a different morphology and behavior despite its apparent similarities in metabolic processes (91). On the other hand, drawing firm conclusions from *in vitro* studies using human spermatozoa is likewise challenging due to methodological differences, such as the use of whole ejaculate *vs* swim-up/density gradient centrifugation-selected cells, or because *in vitro* conditions do not fully reproduce the different microenvironments found in the female reproductive tract.

Human spermatozoa consume less oxygen in seminal fluid than in culture medium (135). Interestingly, oxygen consumption reverts to these lower levels when washed spermatozoa are returned to their own seminal fluid, suggesting the presence of inhibitory factors that suppress the Krebs cycle and OXPHOS. A potential explanation is the presence of the decapacitating factor Zn^2+^, which is a strong inhibitor of the α-ketoglutarate dehydrogenase (KGDH) complex (reported K_i_ of 0.4 µM, with complete inhibition observed at concentrations >3.2 µM (136)), and the ETC through inhibition of the coenzyme Q:cytochrome C (Complex III, reported K_i_ of 100 nM, with complete inhibition observed at concentrations >5 µM (137)) and NADH:ubiquinone oxidoreductase (Complex I, reported K_i_ of 10-50 µM (138)). By contrast, mitochondrial respiration increases during capacitation. Early work by Murdoch and White showed elevated oxygen consumption in capacitating human spermatozoa, an effect enhanced by glucose, fructose and lactate (139). Similar results were obtained by Hereng et al., who showed that human spermatozoa increased oxygen consumption when submitted to capacitating conditions (HCO_3_^-^ and albumin) in the presence of glucose and pyruvate (95). Using labeled pyruvate, Reynolds et al. produced evidence of a progressive metabolic shift towards an increased mitochondrial respiration during capacitation (140). Notably, the capacitation-dependent increase in mitochondrial activity seems to be independent of glycolysis, as an increased mitochondrial membrane potential and increased ROS production are induced by capacitation triggers (HCO_3_^-^, Ca^2+^ and albumin) even in the absence of glucose (94).

Functionally, the inhibition of OXPHOS substantially impairs motility and hyperactivation. Hereng et al. showed that sodium cyanide, a potent inhibitor of complex IV, markedly reduced progressive motility of capacitating human spermatozoa, concomitant with the loss of the capacitation-related protein tyrosine residues phosphorylation cascade and abolition of hyperactivation (96). Rotenone and antimycin A, inhibitors of complexes I and III respectively, produced a similar decline in ATP levels and progressive motility (96). These effects were rescued by pyruvate and methylene blue, an oxidizing agent capable of regenerating NAD^+^ from NADH, indicating that, in addition to ATP production, OXPHOS in human spermatozoa serves an essential role in replenishing the NAD^+^ pools required for glycolysis. Interestingly, pyruvate as the sole energy substrate did not induce human spermatozoa hyperactivation but increased protein tyrosine residues phosphorylation, suggesting that OXPHOS contributes to the activation of capacitation-related signaling pathways despite its kinetics being potentially insufficient to meet the high ATP demand required to sustain hyperactivated motility (96). Supporting these results, Marin-Briggiler et al. showed that rotenone, antimycin A, and carbonyl cyanide 3-chlorophenylhydrazone (CCCP), a mitochondrial uncoupler, induces the total loss of total and progressive motility in energy substrate-deprived (starvation) conditions, effects that were absent when the media contained glucose and pyruvate (141). Overall, current evidence indicates that OXPHOS complements glycolysis in human spermatozoa, with each pathway fulfilling distinct roles: glycolysis provides rapid, localized ATP supply, while OXPHOS supports ATP production, maintains oxidized reducing equivalents for glycolysis, and contributes to the activation of capacitation-related signaling pathways. Both pathways are therefore essential, and their coordinated activity is required for successful capacitation and hyperactivation in human spermatozoa.

It is important to notice that the findings of Marin-Briggiler et al., who showed that ETC inhibitors markedly impair motility in starvation conditions, strongly suggest that the mitochondria can metabolize endogenous energy substrates to sustain motility independently of glycolysis (141). Conversely, Williams and Ford showed that 2-DG also reduces motility in starvation conditions, implying that some endogenous energy substrates require glycolytic enzymes and/or intermediates for their utilization (66). Consistent with these observations, Carrageta et al. found that human spermatozoa can maintain motility for at least 6h in the absence of exogenous energy sources (94). Taken together, these data support the hypothesis that human spermatozoa undergo multiple additional metabolic pathways to meet their energetic demands.

## 3 Additional metabolic pathways fueling motility and capacitation in human spermatozoa

**Figure 3:**
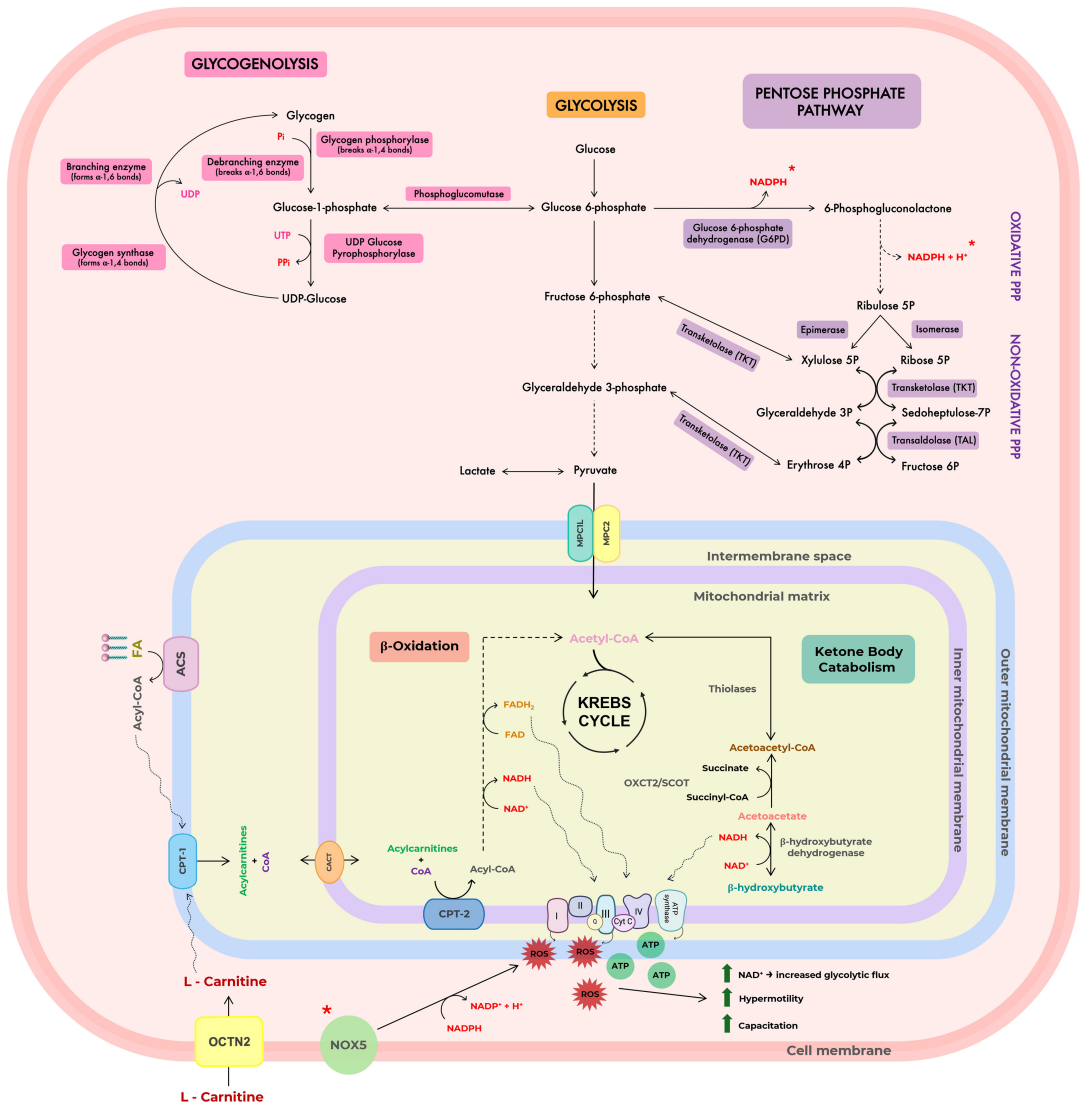
Schematic overview of alternative metabolic pathways contributing to energy production in human spermatozoa. In addition to glycolysis and oxidative phosphorylation (OXPHOS), spermatozoa possess enzymatic machinery supporting glycogen metabolism, allowing stored glycogen to be mobilized into glucose 6-phosphate and fed into glycolysis. The pentose phosphate pathway (PPP) is responsible for producing NADPH, activating reactive oxygen species (ROS) production by NADPH oxidases. Lipid metabolism pathways, including mitochondrial β-oxidation of fatty acids, provide acetyl-CoA and reducing equivalents (NADH and FADH_2_) that fuel the Krebs cycle and OXPHOS. Furthermore, ketone body catabolism offers additional oxidative substrates, contributing with acetyl-CoA to mitochondrial metabolism. Together, these pathways highlight the metabolic flexibility of human spermatozoa, enabling sustained ATP production and functional competence under fluctuating energetic and environmental conditions. *NADPH produced in PPP is used by NOX5 for ROS production. ACS, acyl-CoA synthetase; ATP, adenosine triphosphate; CACT, carnitine–acylcarnitine translocase; CPT-1 - carnitine palmitoyltransferase 1; CPT-2 - carnitine palmitoyltransferase 2; Cyt C, cytochrome c; FAD, flavin adenine dinucleotide; FADH_2_, reduced flavin adenine dinucleotide; MPC1L, mitochondrial pyruvate carrier 1-like; MPC2, mitochondrial pyruvate carrier 2; NAD^+^, oxidized nicotinamide adenine dinucleotide; NADH, reduced nicotinamide adenine dinucleotide; NADP^+^, oxidized nicotinamide adenine dinucleotide phosphate; NADPH, reduced nicotinamide adenine dinucleotide phosphate; NOX 5, NADPH oxidase 5; OXCT2/SCOT, succinyl-CoA:3-oxoacid CoA transferase; Q, coenzyme Q10.

Human spermatozoa exhibit considerable metabolic plasticity. While current evidence establishes glycolysis and OXPHOS as the main ATP-producing pathways that support capacitation and hyperactivation, several studies suggest the existence of complementary metabolic pathways that may modulate these processes in human spermatozoa (**Table 4**, **Figure 3**).

**Table 4:** Molecular and catalytic properties, subcellular location, and putative role in capacitation and hyperactivation of enzymes belonging to amino acid metabolism, ketolysis, pentose-phosphate pathway, and glycogen metabolism pathways in human spermatozoa.

Metabolic pathway	Key enzymes	Subcellular location	Source of evidence	Molecular and catalytic properties	Putative role in capacitation and hyperactivation	Functional validation
Amino acid Metabolism	L-amino acid oxidase (LAAO)	LAOO is present in the acrosome region and midpiece (148)	Immunofluorescence (148)	Conversion of L-amino acids (mainly phenylalanine), water, and oxygen into α-keto acids, ammonia, and hydrogen peroxide	Use L-amino acids as substrate for ROS production, thereby supporting capacitation-related signaling pathways and acrosome reaction	Treatment with phenylalanine stimulated hydrogen peroxide production in a dose-dependent manner, which promoted capacitation (increased phosphotyrosine residues levels) and acrosome reaction. These effects were absent when spermatozoa were incubated with catalase (148)
Ketolysis	Mitochondrial β-hydroxybutyrate dehydrogenase (BDH1)	BDH1 is located in the midpiece (188)	Immunofluorescence (188)	Catalyzes the oxidation of β-hydroxybutyrate into acetoacetate, the initial step of ketolysis	Produce acetyl-CoA for the Krebs cycle, sustaining OXPHOS	No available data
	Testis-specific succinyl-CoA:3-oxoacid coenzyme A transferase (SCOT-t)	SCOT-t is located in the midpiece and to a lesser extent in the head (149)	Western Blotting and Immunofluorescence (149)	Catalyzes the transfer of coenzyme A from succinyl-CoA to acetoacetate to produce acetoacetyl-CoA and succinate		β-hydroxybutyrate sustains the progressive motility of capacitated human spermatozoa, an effect that is absent when SCOT is inhibited (152)
Pentose-phosphate pathway (PPP)	Glucose-6-phosphate dehydrogenase (G6PD)	Evidence suggests G6PD is located in the equatorial/subequatorial region of the head and midpiece (153)	Fluorimetric enzymatic activity assay (153)	Oxidation of glucose-6-phosphate into 6-phosphogluconolactone with the production of NADPH, the initial step of PPP	Regulate redox homeostasis by producing NADPH, which supports ROS production by NADPH oxidases and sustains the glutathione antioxidant system by promoting glutathione reduction	Controlled oxidative stimuli induces PPP-related NADPH production to reduce glutathione and regenerate the glutathione antioxidant system (154) G6PD inhibition by DHEA reduces NOX-related superoxide production, leading to capacitation arrest (155)
Glycogen metabolism	Glycogen synthase (GYS1)	GYS1M (muscle-type) was not validated in human spermatozoa	Proteomics data (126)	Rate-limiting enzyme responsible for glycogen synthesis, by transferring glucose molecules from UDP-glucose onto the end of a pre-existing glycogen chain	Capacitation-induced glycogenolysis may provide glucose residues to sustain glycolysis	No available functional data *Functional glycogen metabolism has been demonstrated in dog spermatozoa, as glycogenolysis is stimulated under starvation conditions* (167). *Additionally, GYS1 activity appears to depend on glucose and fructose availability* (168)
	Glycogenin-1 (GYG1)	GYG1 was not validated in human spermatozoa		Glycosyl transferase that initiates glycogen biosynthesis by promoting its autoglycosilation using UDP-glucose until forming an oligosaccharide composed of 8 glucose residues		
	Glycogen phosphorylase (PYG)	PYG1M (muscle-type), PYG1B (brain-type), and PYG1L (liver-type) were not validated in human spermatozoa		Responsible for glycogenolysis by breaking down glycogen into glucose-1-phosphate		
	Glycogen debranching enzyme (GDE)	GDE was not validated in human spermatozoa		Responsible for glycogenolysis by removing branches and ensuring efficient glucose release		

The first proteomics studies detected several enzymes previously unidentified in human spermatozoa, suggesting that certain metabolic pathways are likely active despite the lack of mechanistic data (125, 142). For instance, amino acid metabolism emerges repeatedly in proteomics and metabolomics studies, although direct functional data remain sparse. Alterations in amino acid metabolism are frequently observed in asthenozoospermia cohorts (16, 17, 143, 144), however, most studies emphasize antioxidant or cryoprotective properties rather than their potential metabolic contributions (145–147). The clearest functional data comes from the identification of L-amino acid oxidase (LAAO) in human spermatozoa. According to Houston et al., LAAO is present in the midpiece and acrosome region of human spermatozoa, where it converts L-amino acids, water, and oxygen into α-keto acids, ammonia, and hydrogen peroxide (H_2_O_2_) (148). When stimulated by aromatic amino acids, mainly phenylalanine and tryptophan, LAAO increased the production of hydrogen peroxide, which resulted in increased protein tyrosine phosphorylation and acrosomal exocytosis. Confirming that these effects are mediated by an LAAO-ROS axis, incubation with catalase reverted the capacitation and acrosome reaction-related effects (148). Although several amino acids can, in principle, be catabolized to Krebs cycle intermediates and generate reducing equivalents for OXPHOS, direct functional evidence that amino acid catabolism fuels human spermatozoa bioenergetic requirements demands further research.

Ketolysis is another metabolic pathway that is may be active in human spermatozoa, as suggested by proteomics studies (125, 142). Ketolysis, as shown in **Figure 3**, is initiated by the oxidation of β-hydroxybutyrate into acetoacetate by the mitochondrial β-hydroxybutyrate dehydrogenase. Acetoacetate is then converted into acetoacetyl-CoA by the succinyl-CoA:3-oxoacid coenzyme A transferase (SCOT), which mediates the transfer of coenzyme A from succinyl-CoA to acetoacetate. A testis-specific SCOT isozyme was identified (SCOT-t), displaying stronger immunostaining in the midpiece and fainter in the head of human spermatozoa (149). Acetoacetyl-CoA is finally cleaved by a group of ubiquitous enzymes known as thiolases, generating two molecules of acetyl-CoA that subsequently enter the Krebs cycle (150, 151). Although direct evidence is scarce, available functional data indicate that ketolysis may play a role in capacitated human spermatozoa. Pappalardo et al. showed that capacitated human spermatozoa, but not uncapacitated, can metabolize β-hydroxybutyrate to sustain progressive motility, an effect that was inhibited by the SCOT inhibitor aceto-hydroxamic acid (152). However, no direct association between ketolysis and hyperactivated motility was identified, highlighting that the role of this metabolic pathway, particularly in physiologically relevant conditions, requires further research.

Compared to amino acids and ketone bodies, stronger evidence supports the involvement of additional carbohydrate pathways, such as pentose-phosphate or glycogen pathways, and lipid pathways in capacitation and hyperactivation. In terms of energy-generating pathways, evidence for the existence of endogenous energy sources capable of sustaining human spermatozoa motility under starvation conditions, such as glycogen and fatty acids β-oxidation, was reported in multiple *in vitro* studies (69, 94, 141). Although such conditions are unlikely to occur physiologically, these metabolic pathways may operate as complementary mechanisms to meet the high ATP demands associated with capacitation and hyperactivation.

### 3.1 Pentose-phosphate pathway involvement in the capacitation of human spermatozoa

The pentose-phosphate pathway (PPP) (**Figure 3**), which runs in parallel to glycolysis in the cytosol, begins with the oxidation of glucose-6-phosphate into 6-phosphogluconolactone by glucose 6-phosphate dehydrogenase (G6PD). PPP has the main function of producing reducing equivalents (reduced nicotinamide adenine dinucleotide phosphate – NADPH) and pentoses. Early histochemical studies detected G6PD activity and NADPH production in human spermatozoa, mainly in the midpiece and equatorial/subequatorial region of the head (153). Although data are scarce, evidence suggests two important roles for PPP-derived NADPH in human spermatozoa: fueling the glutathione antioxidant system, as a cofactor for glutathione reductase (154), and as a substrate for NADPH oxidase (NOX), producing signaling levels of free radical superoxide (O_2_^-^) (155).

William and Ford demonstrated that the PPP is activated when human spermatozoa are submitted to a moderate oxidative stimulus, supplying the NADPH required to reduce glutathione and thereby regenerating the glutathione antioxidant defense system (154). Human spermatozoa are highly susceptible to oxidative stress, which is considered a major cause of infertility (156–158); nevertheless, ROS production is a double-edged sword for human spermatozoa as ROS signaling is required for capacitation and hyperactivation through activation of adenylyl cyclase, activation of the cAMP-PKA pathway, and subsequent increase in protein tyrosine residue phosphorylation (159–162). One of the main ROS producers in human spermatozoa is NOX, which uses NADPH as a substrate to produce the free radical superoxide. NOX5 is the only NADPH oxidase identified in human spermatozoa (163). Immunolocalization shows that NOX5 shares the subcellular location with G6PD, being mainly present in the midpiece and acrosome region (163). Mechanistically, the association PPP-NOX was demonstrated by Miraglia et al., who showed that PPP activity increases during human spermatozoa capacitation while its inhibition, using the uncompetitive G6PD inhibitor dehydroepiandrosterone (DHEA), reduces superoxide production by NOX and blocks capacitation (155). Based on its location, the PPP-NOX axis was also hypothesized to play a role in the acrosome reaction. Although never validated in human spermatozoa, this hypothesis was partially confirmed in mice, as exogenous NADPH supports gamete fusion in the absence of glucose, thereby implicating the PPP-NOX axis in the process (164).

### 3.2 Evidence for glycogen metabolism in human spermatozoa

Glycogen has been proposed as a potential endogenous energy reserve that could support human spermatozoa motility, a hypothesis supported by the observation that 2-DG reduces motility under starvation conditions (66). Although glycogen granules have not been formally reported, proteomics studies have detected key enzymes involved in glycogen metabolism in human spermatozoa, including glycogen synthase, glycogenin-1, glycogen phosphorylase (liver, brain, and muscle isoforms), and glycogen debranching enzyme (126, 165), suggesting that this pathway may be active. Interestingly, comparative studies across mammalian species indicate that glycogen metabolism in spermatozoa is species-dependent: glycogen staining was negative in rodents (166), whereas it was successfully detected in the head and midpiece of spermatozoa from dogs, boar, stallion, and ram (167). Among these, a functional glycogen metabolism has been demonstrated only in dog spermatozoa. Ballester et al. demonstrated that glycogen is consumed in starvation conditions, whereas increasing extracellular glucose or fructose levels leads to a corresponding increase in intracellular glycogen levels (167). Consistently, Palomo et al. showed that glycogen synthase activity is dependent on glucose and fructose availability (168). In a follow-up study using an isotopically labeled lactate tracer, Albarracín et al. showed that dog spermatozoa can synthesize glycogen from lactate via gluconeogenesis (169). Moreover, inhibition of pyruvate carboxylase with phenylacetic acid reduced protein tyrosine residue phosphorylation, implicating this pathway in capacitation (169). Despite these findings, orthogonal validation of the enzymes expression and functional data on glycogen metabolism remain inexistent in human spermatozoa (170).

### 3.3 Fatty acids β-oxidation: the likely hidden endogenous energy source?

The concept that mammalian spermatozoa can oxidize fatty acids from endogenous phospholipids in the absence of glycolysable substrates is not new. As early as the 1960s, a study using isotopically labeled fatty acid tracers reported that spermatozoa from various mammalian species can oxidize fatty acids (171). In human spermatozoa, the incorporation of extracellular fatty acids into membrane phospholipids has been demonstrated (172), however, the evidence for fatty acid catabolism is scarce.

As demonstrated in **Figure 3**, fatty acid catabolism occurs predominantly through mitochondrial β-oxidation, a pathway that produces acetyl-CoA, which subsequently enters the Krebs cycle to generate reducing equivalents that fuel OXPHOS. Mitochondrial β-oxidation is usually favored under aerobic conditions and during states of increased energy demand or reduced ATP availability. The beginning of β-oxidation, however, occurs in the cytosol, where fatty acids must be activated before being transported into the mitochondria. Fatty acids activation is catalyzed by acyl-CoA synthetase (ACS, also known as acyl-CoA ligase) in an ATP-dependent, two-step process that converts fatty acids into fatty acyl-CoAs using coenzyme A (173). Since the mitochondrial membrane is impermeable to fatty acyl-CoAs, their entry into the mitochondrial matrix requires conjugation to L-carnitine in a process known as the carnitine shuttle. Carnitine shuttle is the rate-limiting step of β-oxidation, initiated by the outer mitochondrial membrane-bound carnitine palmitoyl transferase 1 (CPT-1) which transfers the acyl group from CoA to L-carnitine to generate acylcarnitine and free CoA. Acylcarnitine is then translocated across the inner mitochondrial membrane by the carnitine acylcarnitine translocase (CACT). Within the matrix, the inner mitochondrial membrane-bound carnitine palmitoyl transferase 2 (CPT-2) cleaves the L-carnitine from the acylcarnitine, regenerating acyl-CoA (174). In the meantime, the regenerated acyl-CoA is then ready to enter β-oxidation, while L-carnitine returns to the cytoplasm to start another cycle via CACT (174). At the first step of β-oxidation, acyl-CoAs are oxidized to *trans*-Δ^2^-enoyl-CoA by an acyl-CoA dehydrogenase (ACAD) in a FAD-dependent reaction. The canonical straight-chain ACADs can be categorized into four distinct groups, based on their specificity: very long-chain ACAD (ACADVL), long-chain ACAD (ACADL), medium-chain ACAD (ACADM), and short-chain ACAD (ACADS). In the second step, *trans*-Δ²-enoyl-CoA is hydrated to L-3-hydroxyacyl-CoA by enoyl-CoA hydratase (ECHS1), followed by its oxidation to 3-ketoacyl-CoA by 3-hydroxyacyl-CoA dehydrogenase (HADH) and using NAD^+^ as an electron acceptor. At the final step, 3-ketoacyl-CoA thiolase (ACAA2) catalyzes the thiolysis of 3-ketoacyl-CoA into acetyl-CoA, which may feed the Krebs cycle, and a fatty acyl-CoA minus two carbons that will undergo a new cycle until all the carbons are turned into acetyl-CoA (175). In the case of very-long- and long-chain fatty acids, the mitochondrial trifunctional protein (MTP), catalyzes the last three steps of β-oxidation (176, 177).

Similar to PPP and glycogen metabolism, early evidence for lipid metabolism in human spermatozoa came from proteomics studies (**Table 5**). For instance, Amaral et al. reported that among metabolism and energy production-related proteins, 24% are implicated in lipid metabolism, including fatty acid β-oxidation, carnitine shuttle, ketone body catabolism, glycerol degradation, and phospholipid and triglyceride biosynthesis (125). Moreover, the observation that ETC inhibitors markedly impair human spermatozoa motility under starvation conditions strongly suggests that mitochondria can oxidize endogenous substrates to sustain motility independently of glycolysis, with β-oxidation as the most likely candidate (141). However, protein-level and mechanistic validation are still at its infancy. Among the known ACS family members, only long-chain fatty acid-CoA ligase 1 (ACSL1) has been identified in the midpiece of human spermatozoa (178). Proteomics datasets suggest that ACSL3 and ACSL5 may also be present, although orthogonal validation has not yet been reported (126). CPT-1 has been identified in the head and midpiece of human spermatozoa (178), whereas CPT-1B and CPT-2 have so far been detected only in proteomics datasets (126). Among the ACADs, only ACADVL and ACADL have been identified in the midpiece of human spermatozoa (178), while ACADM, ACADS, and ACADSB have been reported only in proteomics datasets (125). Similarly, MTP has been identified in the midpiece of human spermatozoa (178), whereas ECSH1, HADH, and ACAA2 have so far been detected in proteomics datasets (125). Mechanistically, early evidence for an active β-oxidation in human spermatozoa came from the fact that etomoxir, a CPT-1 inhibitor that prevents the import of fatty acids into mitochondria, greatly impairs motility and long-term vitality in human spermatozoa incubated in starvation conditions (125). More recently, Li et al. demonstrated that inhibiting the nuclear factor-κB (NF-κB) signaling pathway induced a hyperactivated-like motility in human spermatozoa under starvation conditions, which was associated with an increased β-oxidation flux (178). The motility alterations induced by NF-κB inhibition were associated with reduced intracellular levels of several fatty acids (oleic, adrenic, stearic, and palmitic acid) and increased ATP levels, whereas motility effects were abolished in the presence of etomoxir (178). Overall, these findings indicate that β-oxidation is a metabolic pathway with the ability to sustain human spermatozoa motility, whose flux likely increases during capacitation to assist in the production of reducing equivalents through the Krebs cycle. Nevertheless, further studies are required to clarify this hypothesis and whether β-oxidation may be stimulated as a complementary energetic pathway to sustain hyperactivated motility.

**Table 5:** Molecular and catalytic properties, subcellular location, and putative role in capacitation and hyperactivation of fatty acid oxidation enzymes in human spermatozoa.

Metabolic pathway	Key enzymes	Subcellular location	Source of evidence	Molecular and catalytic properties	Putative role in capacitation and hyperactivation	Functional validation
Fatty acids β-oxidation	Long-chain fatty acid-CoA ligase 1 (ACSL1)	ACSL1 is located in the midpiece (178)	Western Blotting and Immunofluorescence (178)	Catalyzes the conversion of long-chain fatty acids to their active form acyl-CoAs for both synthesis of cellular lipids and degradation via β-oxidation	Pathway that produces acetyl-CoA, which enters Krebs cycle to generate reducing equivalents for OXPHOS-driven ATP and ROS production	Inhibition of CPT-1 by etomoxir decreases motility and long-term vitality in human spermatozoa (125) Inhibition of the NF-κB signaling pathway was associated with a hyperactivated-like motility, which was concomitant with a decrease in intracellular fatty acids levels (oleic, adrenic, stearic, and palmitic acid) and increased ATP levels. These effects were absent when etomoxir (CPT-1 inhibitor) was present (178) *CPT-1B participates in mitochondrial sheath assembly and regulates motility in mouse spermatozoa* (213)
	Long-chain fatty acid-CoA ligase 3 (ACSL3)	Not validated in human spermatozoa	Proteomics data (126)			
	Long-chain fatty acid-CoA ligase 5 (ACSL5)	Not validated in human spermatozoa	Proteomics data (126)			
	Carnitine palmitoyl transferase 1 (CPT-1)	CPT-1A is located in the head and midpiece (178) CPT-1B was not validated in human spermatozoa	CPT-1A was identified by Western Blotting and Immunofluorescence (178) CPT-1B was identified in Proteomics data (126)	Rate-limiting mitochondrial enzyme that catalyzes the conversion of fatty acyl-CoA to acyl-carnitine for mitochondrial transport		
	Carnitine-acylcarnitine translocase (CACT)	Proteomics data suggests that CACT is located in the tail (125)	Proteomics data (125)	Transports the acyl-carnitine across the inner mitochondrial membrane		
	Carnitine palmitoyl transferase 2 (CPT-2)	Proteomics data suggest that CPT-2 is located in the tail (125)	Proteomics data (125)	Catalyzes the conversion of acyl−carnitine back to fatty acyl−CoA in the mitochondrial matrix		
	Very-long-chain acyl-CoA dehydrogenase (ACADVL)	ACADVL is located in the midpiece (178)	Western Blotting and Immunofluorescence (178)	Catalyzes the first step of β-oxidation of very-long-chain fatty acids, producing *trans*-Δ²-enoyl-CoA and FADH_2_		
	Long-chain acyl-CoA dehydrogenase (ACADL)	ACADL is located in the midpiece and base of the head (178)	Western Blotting and Immunofluorescence (178)	Catalyzes the first step of β-oxidation of long-chain fatty acids, producing *trans*-Δ²-enoyl-CoA and FADH_2_		
	Medium-chain acyl-CoA dehydrogenase (ACADM)	Proteomics data suggest that ACADM is located in the tail (125)	Proteomics data (125)	Catalyzes the first step of β-oxidation of medium-chain fatty acids, producing *trans*-Δ²-enoyl-CoA and FADH_2_		
	Short-chain acyl-CoA dehydrogenase (ACADS)	Proteomics data suggest that ACADS is located in the tail (125)	Proteomics data (125)	Catalyzes the first step of β-oxidation of short-chain fatty acids, producing *trans*-Δ²-enoyl-CoA and FADH_2_		
	Mitochondrial trifunctional enzyme (MTP)	MTP subunit α (HADHA) is located in the midpiece (178) Proteomics data that suggests MTP subunit β (HADHB) is located in the tail (125)	HADHA was identified by Western Blotting and Immunofluorescence (178) HADHB was identified by Proteomics data (125)	Multienzyme complex that catalyzes the sequential hydration, second dehydrogenation, and thiolytic cleavage steps (second to fourth step) of long-chain fatty acid β-oxidation, resulting in the production of acetyl-CoA and a shortened acyl-CoA		
	Enoyl-CoA hydratase (ECHS1)	Proteomics data suggests that ECHS1 is located in the tail (125)	Proteomics data (125)	Catalyzes the hydration of *trans*-Δ²-enoyl-CoA to L−3−hydroxyacyl−CoA in medium/short-chain fatty acids (second step)		
	Hydroxyacyl-CoA dehydrogenase (HADH)	Proteomics data suggests that HADH is located in the tail (125)	Proteomics data (125)	Catalyzes the oxidation of L-3-hydroxyacyl-CoA to 3-ketoacyl-CoA producing NADH + H^+^ in medium/short-chain fatty acids (third step)		
	3-ketoacyl-CoA thiolase (ACAA2)	Proteomics data suggests that ACAA2 is located in the tail (125)	Proteomics data (125)	Catalyzes the thiolytic cleavage of 3-ketoacyl-CoA to generate acetyl-CoA and a shortened acyl-CoA in medium/short-chain fatty acids (fourth step)		
Peroxisomal β-oxidation	Peroxisomal Acyl-CoA oxidase 1 (ACOX1)	Not validated in human spermatozoa	Proteomics data (126)	Rate-limiting enzyme that catalyzes the first step of the peroxisomal β-oxidation: oxidation of acyl-CoA to *trans*-Δ²-enoyl-CoA with the production of H_2_O_2_ ACOX1 acts on straight chain acyl-CoAs whereas ACOX3 acts on straight and 2-methyl branched acyl-CoAs	Pathway responsible for chain-shortening substrates that β-oxidation cannot metabolize, including very-long-chain fatty acids, branched-chain fatty acid, and bile acid intermediates May support capacitation through ROS production	No available functional data
	Peroxisomal Acyl-CoA oxidase 3 (ACOX3)	Not validated in human spermatozoa	Proteomics data (126)			
	Peroxisomal multifunctional enzyme (L-bifunctional enzyme or MFP-1)	Not validated in human spermatozoa	Proteomics data (180)	Catalyzes the second and third step of peroxisomal β-oxidation: hydration of *trans*-Δ²−enoyl−CoA to 3−hydroxyacyl−CoA followed by dehydrogenation to 3−ketoacyl−CoA MFP-1 catalyzes and produces L-straight-chain fatty acids. MFP-1 also has Δ^3^, Δ^2^-enoyl-CoA isomerase activity MFP-2 catalyzes and produces D-branched-chain fatty acids. MFP-2 also catalyzes bile acid intermediates metabolism		
	Peroxisomal multifunctional enzyme 2 (D-bifunctional enzyme or MFP-2)	Not validated in human spermatozoa	Proteomics data (126)			
	Peroxisomal 3-ketoacyl-CoA thiolase (ACAA1)	ACAA1 is located in the midpiece (125)	Western Blotting and Immunofluorescence (125)	Catalyzes the cleavage 3-ketoacyl-CoA into acetyl-CoA and a two-carbon shortened acyl-CoA		
	Carnitine O-acetyltransferase (CRAT)	Proteomics data suggest that CRAT is located in the tail (125)	Proteomics data (125)	In peroxisomes, it converts acetyl-CoA into acetyl-carnitine for export, maintaining CoA availability for continuous peroxisomal β-oxidation		
	Peroxisomal carnitine O-octanoyltransferase (CROT)	Not validated in human spermatozoa	Proteomics data (126)	In peroxisomes, converts medium- and branched-chain acyl-CoA into acyl-carnitines for export		
Peroxisomal α-oxidation	Phytanoyl-CoA dioxygenase (PHYH)	PHYH was not validated in human spermatozoa	Proteomics data (180)	Catalyzes the Fe^2+^- and O_2_-dependent α-hydroxylation of phytanoyl-CoA to 2-hydroxyphytanoyl-CoA, the rate-limiting step of α-oxidation	Pathway that removes one carbon from β-methyl-branched fatty acids, enabling subsequent β-oxidation	No available functional data
	2-hydroxyacyl-CoA lyase 1 (HACL1)	HACL1 was not validated in human spermatozoa	Proteomics data (126)	Catalyzes the TPP-dependent cleavage of 2-hydroxyacyl-CoA into a fatty aldehyde and formyl-CoA		
	Long-chain aldehyde dehydrogenase (ALDH3A2)	ALDH3A2 was not validated in human spermatozoa	Proteomics data (126)	Catalyzes the NAD(P)^+^-dependent oxidation of long-chain aliphatic aldehydes into fatty acids		

Surprisingly, even though spermatozoa are believed to be a cell devoid of peroxisomes, proteomics studies were able to identify 70% of all known peroxisomal proteins in human spermatozoa (179). Among those, data suggest the presence of a complete enzymatic machinery for peroxisomal β-oxidation in human spermatozoa, evidenced by the detection of peroxisomal acyl-CoA oxidase 1 and 3 (ACOX1 and ACOX3), which catalyzes the first and rate-limiting step by oxidizing acyl-CoA to *trans*-Δ²-enoyl-CoA with the production of H_2_O_2_, peroxisomal multifunctional enzyme 1 and 2 (MFP-1 and MFP-2), which catalyzes the hydration of *trans*-Δ²-enoyl-CoA to 3-hydroxyacyl-CoA (second step) followed by the dehydrogenation to 3-ketoacyl-CoA (third step), and, finally, peroxisomal 3-ketoacyl-CoA thiolase (ACAA1), which cleaves 3-ketoacyl-CoA into acetyl-CoA and a two-carbon shortened acyl-CoA (126, 180). Despite being devoid of peroxisomes, carnitine O-acetyltransferase (CRAT) and carnitine O-octanoyltransferase (CROT), which convert acetyl-CoA into acetyl-carnitine and medium- and branched-chain acyl-CoA into acyl-carnitine for peroxisome export, respectively, were also detected in proteomics datasets of human spermatozoa (125, 126). Although further protein-level and functional validation are required, peroxisomal β-oxidation could be an additional source of shorter acyl-CoAs for mitochondrial β-oxidation, acetyl-CoA and ROS in human spermatozoa, thereby contributing to the redox signaling that supports capacitation, membrane remodeling, and the acquisition of hyperactivated motility. In addition, the fact that enzymes belonging to peroxisomal α-oxidation were also detected in proteomics datasets, including phytanoyl-CoA dioxygenase (PHYH), 2-hydroxyacyl-CoA lyase 1 (HACL1), and long-chain aldehyde dehydrogenase (ALDH3A2) (125, 126, 180), further suggests that human spermatozoa may also be able to metabolize β-methyl-branched fatty acids. Nevertheless, protein-level and functional validation remain to be disclosed in future research.

## 4 Conclusions and future perspectives

Current evidence indicates that the capacitation and hyperactivation of human spermatozoa rely predominantly on glycolysis and OXPHOS. These pathways fulfil distinct but interdependent roles: glycolysis provides a rapid, spatially localized ATP supply in the flagellum, whereas OXPHOS complements with bulk ATP production, regenerates reducing equivalents required for continued glycolytic flux, and contributes to the activation of capacitation-associated signaling pathways. Both pathways are therefore essential, and their coordinated activity is required for successful capacitation and hyperactivation in human spermatozoa.

In addition to these core bioenergetic pathways, human spermatozoa possess a high level of metabolic flexibility and can, when/if necessary, exploit alternative energy substrates, including those from endogenous energy reserves, to meet their high energetic demands. This metabolic flexibility suggests a high adaptability to the diversity of microenvironments found throughout the female tract. Nevertheless, there are still several significant questions that remain unanswered. Further research should delineate the identity, regulation, and physiological significance of endogenous energy reserves in human spermatozoa and how such reserves may contribute to capacitation, hyperactivation, and fertilization competence. Among those, fatty acids β-oxidation deserves particular attention, since lipids can be used as an endogenous energy source but are also enriched in the female reproductive tract, particularly in the follicular fluid (181, 182). Methodological approaches that combine high-resolution metabolomics with stable isotope tracing, live-cell metabolic imaging, and extracellular flux analysis will be essential to resolve the metabolic fluxes governing the metabolic flexibility that characterizes human spermatozoa. A deeper mechanistic understanding of human spermatozoa’s metabolic flexibility will, undoubtedly, provide novel biomarkers to understand previous idiopathic infertility and recurrent pregnancy loss cases (183), as well as novel therapeutic targets for medically assisted reproduction.

## Glossary

2-DG: 2-deoxyglucose
ACOX: acyl-CoA oxidase
ACS: acyl-CoA synthetase
ADP: adenosine diphosphate
AK: adenylate kinase
AKAPs: A-kinase anchoring proteins
ALDOA: aldolase A
AMP: adenosine monophosphate
ATP: adenosine triphosphate
CABYR: calcium-binding protein CPB86-IV
CACT: carnitine acylcarnitine translocase
CatSper: spermatozoa cation channel
CCCP: carbonyl cyanide 3-chlorophenylhydrazone
CK: creatine kinase
COX6B2: cytochrome c oxidase subunit 6B2
cAMP: cyclic AMP
CPT-1: carnitine palmitoyl transferase 1
CPT-2: carnitine palmitoyl transferase 2
DHAP: dihydroxyacetone phosphate
DHEA: dehydroepiandrosterone
DNA: deoxyribonucleic acid
ENO1: enolase-1
ENO4: enolase-4
ETC: electron transfer chain
FAD: flavin adenine dinucleotide
FADH_2_: reduced flavin adenine dinucleotide
G6PD: glucose-6-phosphate dehydrogenase
GAPDH: glyceraldehyde-3-phosphate dehydrogenase
GAPDHS: sperm-specific glyceraldehyde-3-phosphate dehydrogenase
GLUTs: glucose-facilitating transporters
HK1S: sperm-specific type 1 hexokinase
Hv1: voltage-gated proton channels
KGDH: α-ketoglutarate dehydrogenase
Ki: inhibition constant
LAAO: L-amino acid oxidase
LDH: lactate dehydrogenase
LDHC: sperm-specific lactate dehydrogenase
MPC1/MPC2: mitochondrial pyruvate carrier 1 and 2
MPC1L: mitochondrial pyruvate carrier 1-like
NAD^+^: oxidized nicotinamide adenine dinucleotide
NADH: reduced nicotinamide adenine dinucleotide
NBC: sodium bicarbonate cotransporters
NF-κB: nuclear factor-κB
NOX: NADPH oxidase
OXPHOS: oxidative phosphorylation
PDH: pyruvate dehydrogenase
PDHA2: sperm-specific E1α subunit of the pyruvate dehydrogenase complex
PDK: pyruvate dehydrogenase kinase
PDP: pyruvate dehydrogenase phosphatase
PFK: phosphofructokinase
PGK2: phosphoglycerate kinase-2
pHi: intracellular pH
PK: pyruvate kinase
pO_2_: oxygen partial pressure
PPP: pentose-phosphate pathway
PTMs: post-translational modifications
ROS: reactive oxygen species
SCOT: succinyl-CoA,3-oxo acid coenzyme A transferase
SGLTs: sodium-coupled glucose transporters
TCA cycle: tricarboxylic acid cycle
ZP: *zona pellucida*
